# Strategy to simulate and fit 2D grazing-incidence small-angle X-ray scattering patterns of nanostructured thin films

**DOI:** 10.1107/S1600576723006520

**Published:** 2023-08-16

**Authors:** Florian A. Jung, Christine M. Papadakis

**Affiliations:** a TUM School of Natural Sciences, Physics Department, Soft Matter Physics Group, Technical University of Munich, James-Franck-Straße 1, Garching 85748, Germany; Australian Centre for Neutron Scattering, ANSTO, Australia

**Keywords:** grazing-incidence small-angle X-ray scattering, GISAXS, distorted-wave Born approximation, nanostructured thin films, simulations, data analysis

## Abstract

Grazing-incidence small-angle X-ray scattering (GISAXS) is a widely used method for the characterization of the nanostructure of supported thin films and enables time-resolved *in situ* measurements. The 2D scattering patterns contain detailed information about the nanostructures within the film and at its surface. However, this information is distorted not only by the reflection of the X-ray beam at the substrate–film interface and its refraction at the film surface but also by scattering of the substrate, the sample holder and other types of parasitic background scattering. In this work, a new, efficient strategy to simulate and fit 2D GISAXS patterns that explicitly includes these effects is introduced and demonstrated for (i) a model case nanostructured thin film on a substrate and (ii) experimental data from a microphase-separated block copolymer thin film. To make the protocol efficient, characteristic linecuts through the 2D GISAXS patterns, where the different contributions dominate, are analysed. The contributions of the substrate and the parasitic background scattering – which ideally are measured separately – are determined first and are used in the analysis of the 2D GISAXS patterns of the nanostructured, supported film. The nanostructures at the film surface and within the film are added step by step to the real-space model of the simulation, and their structural parameters are determined by minimizing the difference between simulated and experimental scattering patterns in the selected linecuts. Although in the present work the strategy is adapted for and tested with *BornAgain*, it can be easily used with other types of simulation software. The strategy is also applicable to grazing-incidence small-angle neutron scattering.

## Abstract 2

A strategy to simulate and fit 2D grazing-incidence small-angle X-ray scattering patterns of supported, nanostructured soft-matter thin films using the distorted-wave Born approximation is introduced. The different scattering contributions of the nanostructure, surface roughness and background scattering are treated separately and are adjusted step by step. To minimize calculation efforts, 1D line cuts are chosen, in which the scattering is predominantly attributed to one of the contributions, and the parameters found are used in the subsequent steps. Hence, separate measurements of the bare substrate are beneficial.

## 1. Introduction

Since the grazing-incidence small-angle X-ray scattering (GISAXS) method was first reported (Levine *et al.*, 1989 ▸; Naudon, 1995 ▸), it has become a widely used tool for the structural characterization of nanostructured surfaces and thin films because it is non-destructive and offers access to the structural parameters of surface and buried structures. A wide range of length scales is accessible, ranging from the nano- to the micrometre length scale, and structural information within the film plane and along the surface normal can be obtained simultaneously (Renaud *et al.*, 2009 ▸; Müller-Buschbaum, 2009 ▸; Hexemer & Müller-Buschbaum, 2015 ▸). The compatibility of the GISAXS method with a large number of sample environments enables *in situ* investigations. Systems investigated using GISAXS include, among others, nanostructures from metals or semi-conductors (Renaud *et al.*, 2009 ▸), magnetic layers (Wang *et al.*, 2017 ▸), nanoporous or nanocomposite films (Doshi *et al.*, 2003 ▸; Gibaud *et al.*, 2003 ▸; Lee, Park *et al.*, 2005 ▸; Li *et al.*, 2018 ▸; Alvarez-Fernandez *et al.*, 2020 ▸), surface gratings (Soccio *et al.*, 2015 ▸), layers of colloids or nanoparticles (Ukleev *et al.*, 2017 ▸; Wu *et al.*, 2018 ▸; Saxena & Portale, 2020 ▸; Engström *et al.*, 2020 ▸; Qdemat *et al.*, 2020 ▸; Schaper *et al.*, 2021 ▸), molecular layers (Guennouni *et al.*, 2017 ▸), organic electronics from perovskites or polymers (Müller-Buschbaum, 2018 ▸; Yin *et al.*, 2022 ▸), and block copolymer thin films (Smilgies *et al.*, 2002 ▸; Müller-Buschbaum, 2003 ▸; Kim *et al.*, 2004 ▸; Müller-Buschbaum, Hermsdorf *et al.*, 2004 ▸; Cavicchi *et al.*, 2005 ▸; Lee, Park *et al.*, 2005 ▸; Busch *et al.*, 2007 ▸; Paik *et al.*, 2010 ▸; Di *et al.*, 2012 ▸; Ree, 2014 ▸; Müller-Buschbaum, 2016 ▸; Posselt *et al.*, 2017 ▸; Smilgies, 2021 ▸). Synchrotron radiation affords high time resolution (sub-second), and time-resolved investigations have elucidated growth and restructuring processes in a number of systems, such as the adsorption of sputtered metals on various substrates (Schwartzkopf *et al.*, 2013 ▸), the crosslinking of nanoparticles into superlattices (Maiti *et al.*, 2019 ▸), the structural changes in solar cells during storage and operation (Müller-Buschbaum, 2014 ▸, 2018 ▸; Yang *et al.*, 2020 ▸), and the structural changes during spin-coating of polymer thin films (van Franeker *et al.*, 2017 ▸) and during solvent vapour annealing of block copolymer thin films (Gowd *et al.*, 2010 ▸; Gu *et al.*, 2014 ▸; Posselt *et al.*, 2017 ▸; Smilgies, 2021 ▸).

However, the full potential of GISAXS is not always exploited, because it may be difficult to extract quantitative information from the 2D patterns. The reasons are that the data analysis is challenging and fitting of advanced models is often hampered, because the reflection of the X-ray beam at the film–substrate interface and – in the case of supported thin films – its refraction at the film surface must be taken into account, which is most often done by applying the distorted-wave Born approximation (DWBA) (Sinha *et al.*, 1988 ▸; Rauscher *et al.*, 1995 ▸; Naudon, 1995 ▸; Busch *et al.*, 2006 ▸; Müller-Buschbaum, 2009 ▸; Renaud *et al.*, 2009 ▸). This introduces several complications:

(i) Due to the anisotropic nature of thin films, the recorded 2D scattering patterns *I*(*q_y_*, *q_z_*) (*q_y_* and *q_z_* are the components of the scattering vector in the film plane and along the surface normal) cannot be reduced to a 1D scattering curve *I*(*q*) by azimuthal averaging. Thus, the 2D patterns must be analysed, which is time consuming, especially when fitting complex structural models to the data.

(ii) Background scattering, for example scattering from the substrate, cannot easily be subtracted, and absolute intensity calibrations are not possible.

(iii) The scattering due to surface roughness of the film or the substrate may overlap with scattering from the nano­structure and can often not be neglected (Li *et al.*, 2018 ▸).

Numerous methods to perform the analysis of GISAXS data with reasonable effort have emerged over the years. In cases where Bragg reflections are present, these can be indexed to determine the symmetry of the periodic nano­structures (Gibaud *et al.*, 2003 ▸; Cavicchi *et al.*, 2005 ▸; Lee, Park *et al.*, 2005 ▸; Busch *et al.*, 2007 ▸; Paik *et al.*, 2010 ▸; Ree, 2014 ▸; Jiang, 2015 ▸; Gunkel *et al.*, 2016 ▸; Wang *et al.*, 2017 ▸; Maiti *et al.*, 2019 ▸; Qdemat *et al.*, 2020 ▸; Saxena & Portale, 2020 ▸; Jung *et al.*, 2020 ▸). The *q_z_* positions need to be evaluated within the DWBA, which is straightforward when the critical angle of the film is known (Di *et al.*, 2012 ▸). 1D linecuts through the reflections can be fitted with appropriate peak functions to determine their widths in the *q_y_* and *q_z_* directions. Using the Bragg and the Debye–Scherrer equations allows one to calculate the repeat distances of the nanostructure in the film plane and along the film normal and the grain size of the ordered domains (Smilgies, 2009 ▸). This way, large datasets can be efficiently analysed; however, this method is limited to data with clear Bragg reflections and a certain number of higher-order reflections – which are not always present in soft-matter systems due to their weak order and/or low scattering contrast – and it gives only limited information about the size and shape of the nanodomains or particles, because the influence of the form factor on the position and shape of the Bragg reflections is neglected (Renaud *et al.*, 2009 ▸).

Another strategy is the fitting of a combination of form and structure factors to 1D linecuts at constant *q_z_* (Schaffer *et al.*, 2013 ▸). Since the distortion effects of the DWBA act only on *q_z_*, the much simpler Born approximation (BA) can be used to analyse 1D linecuts. In this way, both the particle size and the spacing can be extracted; however, it needs to be carefully checked whether decoupling of the scattering in the film plane and along the film normal is justified for the given morphology.

Experimentally, an undistorted scattering pattern can be obtained by measuring in grazing-incidence transmission mode (GTSAXS) (Lu *et al.*, 2013 ▸; Xia *et al.*, 2021 ▸; Ji *et al.*, 2022 ▸). In this mode, the edge of the sample is illuminated, which leads to substantial scattering below the sample horizon. This transmission scattering can be analysed within the BA. However, the sample requirement of GTSAXS, namely that the morphology at the edge is representative, may not always be fulfilled.

Recently, a method was proposed to remove the effects of refraction and reflection and to reconstruct an undistorted scattering pattern, which can be analysed within the BA (Liu & Yager, 2018 ▸). Although this method is promising, it has limitations, *e.g.* for films that are far from uniform along the film normal.

It emerges that the experimental and analysis methods described have certain restrictions, such as specific sample requirements, they rely on certain assumptions to simplify the data analysis or they provide only limited access to the structural parameters encoded in the scattering patterns. Ultimately, simulations of 2D GISAXS patterns of the assumed real-space structure within the DWBA are expected to become the predominant way of analysing GISAXS data. Several software packages for simulating 2D GISAXS patterns are available, among them *IsGISAXS* (Lazzari, 2002 ▸), *FitGISAXS* (Babonneau, 2010 ▸), *HipGISAXS* (Chourou *et al.*, 2013 ▸) and *BornAgain* (Burle *et al.*, 2018 ▸; Pospelov *et al.*, 2020 ▸). They have all been successfully used to carry out simulations of 2D GISAXS patterns of (simple) real-space thin film morphologies. This way, the characteristic scattering features in the experimental 2D GISAXS patterns can often be reproduced to a certain extent, allowing the determination of the morphology and the extraction of structural parameters.

However, some challenges remain which hinder broader application. First, choosing a suitable real-space model on the basis of a 2D GISAXS pattern requires a certain understanding of reciprocal space maps and the typical distortion effects within the DWBA. Machine-learning-aided pattern recognition by means of convolutional neural networks is an upcoming tool, which will likely simplify this choice (Liu *et al.*, 2019 ▸; Ikemoto *et al.*, 2020 ▸). Second, even simple real-space models feature a significant number of parameters to adjust, including parameters describing the film itself, such as its thickness and refractive index; parameters describing the surface roughness, such as the root-mean-square roughness and the correlation length; and parameters describing the nanostructure in the film. Third, simulating full 2D GISAXS patterns, especially from large-area detectors, is computationally heavy, which makes adjusting parameters a tedious and time-consuming task. However, it would be desirable to be able to obtain structural information about the sample in quasi-real time during beam time, which would enable the experimenter to use beam time efficiently, especially when carrying out time-resolved measurements (Pandolfi *et al.*, 2018 ▸).

In view of these challenges, in this work, a strategy is developed to simulate and fit 2D GISAXS patterns, addressing points (i)–(iii) mentioned above. The strategy consists of several step. Each step targets a specific contribution to the scattering pattern, and only those parameters that describe this contribution are adjusted. To reduce the computation time, the entire measured 2D GISAXS pattern is not considered, but the adjustment or fitting of parameters is performed by choosing suitable regions of interest (ROIs) of the patterns (*i.e.* regions in reciprocal space). This strategy is not restricted to a certain available simulation software, but is rather a guideline that can be followed using all available simulation software packages. In the present work, we use *BornAgain* (Burle *et al.*, 2018 ▸; Pospelov *et al.*, 2020 ▸) to explain the strategy and to demonstrate the effects of the different contributions to a 2D GISAXS pattern from a nanostructured film on a substrate.

The manuscript is structured as follows: in Section 2, the stepwise strategy is outlined using the example of a representative nanostructured thin film on a substrate. The selection of the ROIs and the fitting procedure are outlined, and each step is discussed in depth. In Section 3, the strategy is applied to a measured 2D GISAXS pattern from a block copolymer thin film featuring a complex inner and surface structure (Jung *et al.*, 2021 ▸), and the possibilities and limitations of the new strategy are discussed.

## 2. Strategy

**Figure 1:**
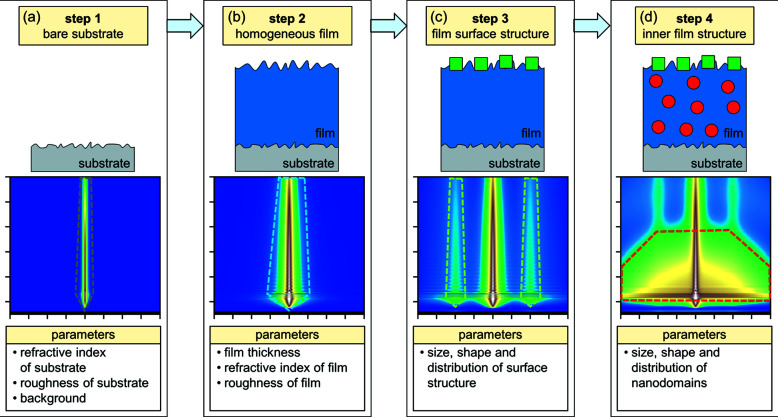
Overview of the stepwise strategy to simulate and fit 2D GISAXS patterns. The upper panels indicate the part of the sample that is simulated in each step of the simulation, and the resulting 2D GISAXS patterns are shown below on a logarithmic intensity scale (see Fig. 2 ▸). Dashed lines in the patterns mark the regions where the respective dominant scattering contributions appear for each step. The main parameters adjusted at each step are listed in the lower panels.

The strategy consists of four main steps and, at each step, more structural features are included in the scattering model (Fig. 1 ▸). To guide the discussion of these steps, we consider a model sample that is inspired by those encountered in block copolymer thin films. It comprises a supported thin film that contains buried, randomly distributed nanospheres in a matrix and ordered cylindrical protrusions on the surface. A detailed description of the model sample and the parameters used for simulation of the 2D GISAXS pattern are given in Section S1 in the supporting information. In brief, the four steps are as follows:

In step 1, the bare substrate is considered. The parameters to be adjusted are the refractive index of the substrate and the substrate surface roughness. In this step, it is also possible to identify and include parasitic scattering features from the instrument or a nanostructured substrate. In step 2, the homogeneous layer of the film is added. The parameters to be adjusted are the thickness, the refractive index and the surface roughness of the film. In step 3, the surface structure of the film is added, which includes parameters describing the size, shape and distribution of the particles. These might, for instance, be protrusions. In step 4, the inner structure of the film is added, which includes parameters describing the size, shape and distribution of the internal nanodomains. Thus, step 4 includes all contributions and is the simulation of the entire sample.

The simulated 2D GISAXS patterns of the model sample at each step are also shown in Fig. 1 ▸. At each step, the most prominent scattering contributions resulting from the structural features considered are indicated by coloured dashed lines. Typical substrates, such as Si wafers, have a low roughness, which gives rise to a narrow vertical streak centred at *q_y_* = 0 in the 2D GISAXS pattern [Fig. 1 ▸(*a*)]. A homogeneous film placed on top of the substrate results in additional scattering, which is mainly due to its surface roughness [Fig. 1 ▸(*b*)]. Thin films from soft matter typically have a higher roughness than the substrate, which leads to a broadening of the vertical rod along *q_y_*. Additionally, fringes along *q_z_* appear, reflecting the finite film thickness. While the contributions of the surface roughness in steps 1 and 2 are dominant near *q_y_* = 0, contributions from the surface structure of the film [Fig. 1 ▸(*c*)] and its inner structure [Fig. 1 ▸(*d*)] in steps 3 and 4 appear mainly at *q_y_* ≠ 0. Often the scattering contrast between the surface particles/protrusions and vacuum is significantly higher than that between the nanodomains in the film and its matrix, and thus, the scattering due to the protrusions is more pronounced.

These stepwise simulations demonstrate that scattering from different structural features appears in different regions in the 2D GISAXS pattern. Thus, it is possible to address the scattering contributions separately by selecting appropriate regions in the scattering pattern and by adjusting the corresponding parameters at each step individually. This protocol leads to a significant reduction of fit parameters in the particular step. In the subsequent steps, the predetermined parameters can be kept fixed. Furthermore, the proposed strategy allows us to distinguish the contributions of the surface roughnesses and the surface structure from those of the inner nanostructure and to identify possible additional contributions. We note that this strategy is not limited to the presented model sample but can be adapted to other types of samples.

The criteria for selecting the ROIs of the 2D scattering patterns and the fitting procedure are described in Section 2.1, and the four steps are discussed in more detail in Sections 2.2–2.5.

### 2.1. Targeting selected regions of the scattering patterns and fitting to experimental data

Comparing and fitting simulated to experimental 2D GISAXS patterns requires an objective function *O* which can be minimized, either by hand or by a suitable minimization algorithm. The most straightforward objective function is the sum of squared residuals:

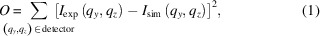

where *I*_exp_(*q_y_*, *q_z_*) and *I*_sim_(*q_y_*, *q_z_*) are the intensities obtained in the experiment and in the simulation, respectively, and the sum in equation (1) runs over all pairs (*q_y_*, *q_z_*), *i.e.* all pixels on the detector. The commonly used detectors have upwards of 10^5^ pixels, which present two challenges:

(i) The intensities of all pixels are recalculated during each iteration of the minimization process, which, even for simpler models, is computationally heavy and time consuming.

(ii) Pixels may have contributions from more than one element of the scattering model, and some pixels may contain irrelevant information, which complicates the minimization process. It is therefore advantageous to limit the minimization to selected ROIs of the scattering pattern (Basioli *et al.*, 2019 ▸).

The appropriate choice of the ROIs depends on the features within the scattering patterns. Still, we identified five major ROIs which give a sufficiently large coverage of most scattering features. Furthermore, the ROIs are chosen such as to allow us to selectively address certain scattering contributions, which is a key advantage of the stepwise strategy. Here, we chose linecuts (*i.e.* 1D intensity profiles) which are constructed by averaging the intensities in either the vertical (horizontal linecuts) or the horizontal (vertical linecuts) direction, yielding 1D intensity profiles at constant *q_z_*, *I*(*q_y_*, *q_z_* = const.) or constant *q_y_*, *I*(*q_y_* = const., *q_z_*), respectively. Thus, horizontal cuts mainly address structural features within the film plane (*q_y_* dependence), whereas vertical cuts address structural features in the direction normal to the film (*q_z_* dependence). Typically, the width of the linecuts (the number of pixels to average) is chosen to be 3–10 pixels. The objective function of the linecuts therefore uses intensities that are averaged over this width:

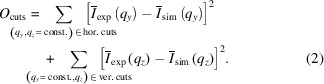

Due to averaging, the noise level of the experimental data is reduced, and the comparison between the experimental and the simulated linecuts is less sensitive to small deviations in *q* calibration.

**Figure 2:**
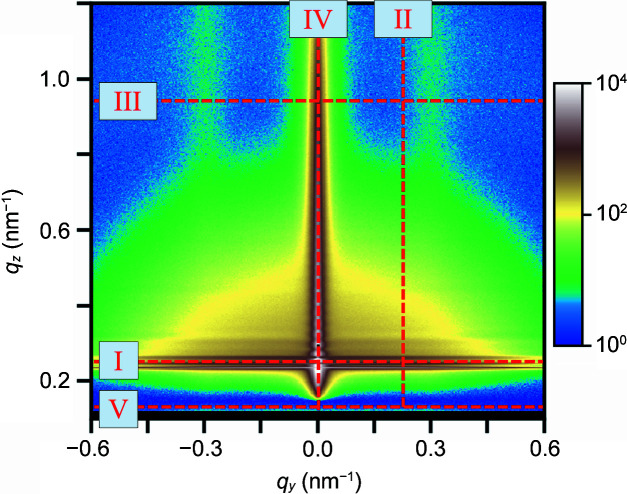
Simulated 2D GISAXS pattern of the model sample (for a detailed description see Section S1 in the supporting information). The logarithmic intensity scale is shown on the right. Linecuts I–V, targeting selected regions of the pattern, are indicated by red lines.

The five types of linecuts of interest include three horizontal and two vertical linecuts. Their positions in the 2D GISAXS pattern of the model sample are indicated in Fig. 2 ▸.

Linecut I is a horizontal linecut lying in the Yoneda band, *i.e.* at *q_z_* values between the critical angles of the substrate and the film, where the scattering intensity is enhanced. Linecut II is a vertical linecut at *q*_y_ ≠ 0. Its *q_y_* position may be varied at each step. Linecut III is a horizontal linecut at *q_z_* values significantly above the Yoneda band. Its *q_z_* position may be varied. Linecut IV is a vertical linecut at *q_y_* = 0. Finally, linecut V is a horizontal linecut at low *q_z_* values, below the Yoneda band.

By comparison with the prominent scattering contributions indicated in Fig. 1 ▸, it is seen that linecut I includes contributions from all features, linecut II is representative of the nanostructure of the film, linecuts III and IV contain mainly contributions of the surface roughnesses, and linecut V is in a region which contains only scattering from the surface structure (step 3) or parasitic scattering (see below) because it is below the Yoneda band.

The use of only a few linecuts in the strategy reduces the computation time immensely. For example, out of the 2.5 × 10^5^ pixels of a 500 × 500 detector, only 1.25 × 10^4^ are needed to define five linecuts with a width of 5 pixels each, which means a reduction of 95%. The reduction is even stronger for detectors with a larger area. Moreover, linecuts do not necessarily have to cover the full length of the detector, but can be limited to a certain section, which further reduces the number of pixels to be simulated. This makes simulation and fitting of complex film structures computationally feasible and allows for a faster sampling of the parameter space.

To minimize the objective function, an optimizer algorithm which searches for a global minimum using constraints should be used. Such optimizers are, for example, accessible in Python through the package *LMFIT* (Newville *et al.*, 2014 ▸). To balance the strong scattering contributions at low *q* against the weaker scattering contributions at high *q*, either a weight term should be introduced in the objective function or the computation should be done on a logarithmic scale.

Complementary to the optimization with an objective function in the linecuts, it is useful to calculate residual plots to obtain an impression of the goodness of fit in the entire 2D pattern. The residual is the relative difference between the experimental and the simulated pattern:

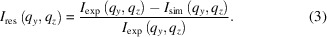

A diverging, continuous colour map gives a good visual impression of the magnitude and sign of the residuals.

### 2.2. Step 1: characterizing the substrate and the background scattering

In the first step, only a bare substrate is included in the simulation. The substrate scattering may be considered as background scattering, but, differently from transmission SAXS, its contribution to the scattering pattern cannot simply be subtracted, because it is altered by the film on top (Lee, Seifert *et al.*, 2005 ▸). The scattering of the substrate is mainly due to its surface roughness, but may, for structured substrates, also include other contributions, for example from islands at the substrate surface or nanopores inside the substrate. In addition to the substrate scattering, other parasitic scattering, *e.g.* from windows, air or the sample holder, may be present as well. Traditionally, all background contributions were handled locally in the data analysis, for example by including empirical decaying functions in the fitting of linecuts (Di *et al.*, 2010 ▸; Sepe *et al.*, 2016 ▸) or by subtracting a local background (Pflüger *et al.*, 2017 ▸). In contrast, simulations allow the construction of a suitable model for the substrate and other background contributions and, subsequently, a model for the thin film on top, *i.e.* explicitly including the background in the simulation instead of subtracting it.

#### 2.2.1. Contributions from the substrate

Models describing the surface roughness of the substrate typically include three parameters, namely the root-mean-square roughness σ_rms,sub_, the lateral correlation length ξ_sub_ and the Hurst parameter *H*_sub_, which are related to the fractal dimension of the surface (Schlomka *et al.*, 1995 ▸). The effects of the roughness parameters on the scattering patterns and linecuts are outlined in Section S3 in the supporting information. It is observed that σ_rms,sub_ affects only the *q_z_* dependence of the roughness scattering, while ξ_sub_ affects the overall intensity of the vertical streak, the *q_z_* dependence of the intensity – namely how far the intensity of the vertical streak extends along *q_z_* – and its *q_y_* width. The value of *H*_sub_ affects the intensity decay of the vertical streak only at large *q_z_* values. It has only a weak effect on its *q_y_* width and hence is of minor importance. Thus, at least two linecuts, one horizontal and one vertical, are needed to analyse the surface roughness scattering with reasonable accuracy (Schlomka *et al.*, 1995 ▸). Of the five linecuts introduced in Fig. 2 ▸, linecuts III and IV are the most suitable for this task (see Section S3 in the supporting information). Linecut IV lies in the scattering plane (*i.e.* *q_y_* = 0) and displays the intensity profile of the vertical streak. Linecut III shows its *q_y_* dependence, but, due to its position at a high value of *q_z_*, it hardly includes any contribution from the nanostructure.

**Figure 3:**
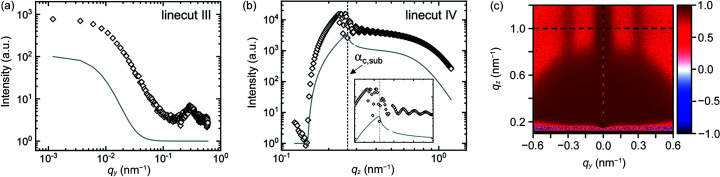
Linecuts (*a*) III at *q_z_* = 1.01 nm^−1^ and (*b*) IV of the simulated 2D GISAXS pattern of the model sample (step 4, open black symbols) and of a simulation including only the substrate with the parameters given in Table S1 of the supporting information (step 1, grey line). The vertical dashed line in (*b*) indicates the critical angle of the substrate, α_c,sub_. The inset in (*b*) shows a close-up of the Yoneda band region from 0.2 to 0.4 nm^−1^. (*c*) Residual plot (*I*_step4_ − *I*_step1_)/*I*_step4_. Horizontal and vertical dashed lines indicate the positions of linecuts III and IV, respectively.

Unfortunately, in many cases, the parameters of the substrate roughness cannot be directly determined from the 2D GISAXS pattern of the nanostructured film. The reason is that the other scattering contributions (steps 2–4 in Fig. 1 ▸) may overlap with that of the substrate roughness. Furthermore, if the film thickness is high, or the film has a high absorption coefficient, the beam is weakened by the film, which leads to a weak signal of the substrate roughness scattering. The former is also the case for the model sample considered here. Fig. 3 ▸ shows linecuts III and IV from simulations of the full film (step 4) and of the corresponding bare substrate (step 1), as well as their residual plot. It emerges that the contribution of the substrate roughness is rather weak, and it cannot be determined by fitting directly to the experimental data. The residual in Fig. 3 ▸(*c*) is positive at all *q*, even at *q_y_* = 0, indicating that the simulation underestimates the scattering intensity by the film. In such cases, it is advisable to determine the roughness parameters of the substrate by GISAXS, X-ray reflectometry (XRR) and/or atomic force microscopy (AFM) measurements on the bare substrate and to use these parameters in the simulation of the 2D GISAXS pattern (Li *et al.*, 2018 ▸).

Comparison of the two linecuts IV implies that the maximum of the bare substrate at the *q_z_* value calculated from its critical angle α_c,sub_ [*i.e.* the Yoneda peak of the substrate, dashed line in Fig. 3 ▸(*b*)] coincides with the upper boundary of the Yoneda band in the linecut of the entire model sample. While this is often the case, it may be that, due to dynamical effects, the position of the Yoneda peak substrate is difficult to locate in the 2D GISAXS patterns of the nanostructured film. In that case, α_c,sub_ can be calculated according to the chemical composition and the mass density of the substrate (Henke *et al.*, 1993 ▸) and can be used as an input parameter for simulations of the nanostructured film. Alternatively, the refractive index of the substrate can be obtained experimentally from complementary GISAXS measurements of a bare substrate and by fitting the position and shape of the Yoneda peak in linecut IV or from complementary XRR measurements on a bare substrate.

In summary, to obtain the substrate parameters, complementary GISAXS measurements on a bare substrate should be carried out, and linecuts III and IV should be evaluated. Alternatively, they can be determined using XRR or AFM, or literature values should be used. Including these parameters in the simulations of the nanostructured film and keeping their values fixed improves the fit quality significantly.

Thus, complementary GISAXS measurements of bare substrates are a powerful way to obtain the surface roughness parameters, σ_rms,sub_, ξ_sub_ and *H*_sub_, and the critical angle (which translates to the refractive index) of the substrate. These parameters can be fixed in subsequent steps. Complementary AFM or XRR may further improve the evaluation of the substrate.

#### 2.2.2. Additional background contributions

Additional parasitic background scattering arises unavoidably from different sources and overlaps with the sample scattering. It includes, for instance, scattering from the X-ray windows or the beamstop, air scattering, and scattering from the sample holder. For example, scattering from the sample holder occurs if the sample is shorter than the footprint of the beam (Pflüger *et al.*, 2017 ▸). Since the length of the footprint is given by *H*_beam_/sin(α_*i*_), where *H*_beam_ is the beam height and α_*i*_ is the incident angle, this situation may occur for small values of α_*i*_, even for small beam heights.

Fortunately, in some cases, it is possible to include these additional background contributions in the simulations. First, the background contributions in the 2D scattering patterns must be identified using the 2D GISAXS pattern of the bare substrate, measured under the same conditions as the sample. It includes all relevant background contributions in addition to the substrate scattering discussed above.

Next, the origin of the background contributions should be identified and described by a suitable scattering model. In the following, we discuss three types of background contributions: direct beam scattering by the holder, *I*_DB_(*q_y_*, *q_z_*); scattering from the rough surface of the holder, *I*_sur_(*q_y_*, *q_z_*); and a *q*-independent, constant background, *I*_cbg_, arising from other sources such as air scattering or an inherent detector background.

Since these different types of background scattering arise from macroscopically separated scatterers, *i.e.* their mutual distance is larger than the coherence length of the beam, the recorded intensities can be written as an incoherent sum of the sample (*i.e.* the substrate and the film) and background contributions:

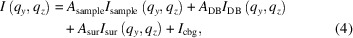

where *A*_sample_, *A*_DB_ and *A*_sur_ are the amplitudes of each contribution, which depend on the fraction of beam intensity giving rise to them. The fact that *I*_DB_(*q* *_y_*, *q* *_z_*) and *I*_sur_(*q* *_y_*, *q* *_z_*) are added incoherently may imply that these terms can be subtracted from the overall scattering intensity. However, as discussed above, this is not necessarily the case, since the beam may have interacted with the sample and may thus have undergone refraction or reflection before being scattered by the background scatterers. It is therefore necessary to include these contributions in the simulation of the substrate and the sample. We note that there may be many other background contributions in addition to those discussed above, *e.g.* X-ray streaks from the edge of the sample or the sample holder. In the case where such contributions are present, a suitable model must be found to describe them. However, the general approach of identifying and modelling them remains the same.

*I*_cbg_ can be easily determined from the asymptotic flattening of the intensities at high *q*. Linecuts I or III may be used for that purpose.

**Figure 4:**
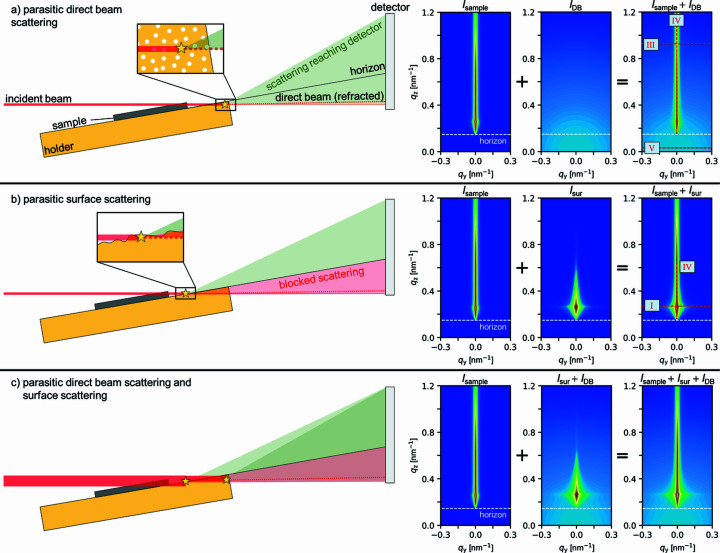
Schematic of potential parasitic background contributions and associated representative simulated 2D GISAXS patterns of (left to right) the sample, background contribution and their sum. The patterns are shown on a logarithmic intensity scale. The scattering events of interest are indicated by stars in the schematics. For illustrative purposes, the dimensions in the schematics are not drawn to scale. The sample is a bare substrate with the same parameters as for the construction of the model sample (Table S1). The same constant background *I*_cbg_ = 1 was used. The horizon (exit angle α_f_ = 0) is marked in the 2D GISAXS patterns by a horizontal dashed white line. (*a*) Parasitic direct beam scattering of nanopores with a radius of 3 nm. (*b*) Parasitic surface roughness scattering from the sample holder with σ_rms,holder_ = 5 nm, ξ_holder_ = 100 nm, *H*_holder_ = 0.5, δ_holder_ = 4.5 × 10^−6^ and β_holder_ = 6 × 10^−8^. (*c*) Combined effect of the scenarios shown in (*a*) and (*b*). The positions of relevant linecuts to identify and adjust the background contributions are indicated with red dashed lines in the right-most patterns. All linecuts are shown in Fig. S3.

Direct beam scattering *I*_DB_(*q* *_y_*, *q* *_z_*) may occur for small incident angles α_i_ or when the direct beam is not fully blocked by the substrate and/or sample holder, allowing transmission scattering to reach the detector below the horizon [Fig. 4 ▸(*a*)]. Transmission scattering is especially severe for GISANS (Kyrey *et al.*, 2021 ▸). Scattering below the horizon is thus a sign of background contributions associated with the direct beam. Examples of this type of scattering are nanopores in the sample holder material or scattering from windows downstream of the sample. The scattering is essentially a SAXS signal, *i.e.* it is isotropic and centred around the direct beam and can therefore be evaluated within the BA. When calculating the direct beam scattering, refraction of the direct beam at the film surface and the substrate surface must be considered (Lu *et al.*, 2013 ▸). Since other contributions are absent below the horizon, linecut V is used to identify and characterize the direct beam scattering. In Fig. 4 ▸(*a*), the effect of direct beam scattering of randomly distributed, spherical nanopores with a radius of 3 nm is shown, namely a weakly decaying additional diffuse intensity contribution.

*I*_sur_(*q_y_*, *q_z_*) denotes the parasitic scattering due to the roughness of a surface other than the substrate surface, for example that of the sample holder, whose roughness is typically significantly higher than that of the sample [Fig. 4 ▸(*b*)]. As an example, the 2D GISAXS pattern of a sample holder surface with high roughness (σ_rms,holder_ = 5 nm, ξ_holder_ = 100 nm, *H*_holder_ = 0.5) and a refractive index similar to the substrate is shown in Fig. 4 ▸(*b*). As outlined in Section S3 in the supporting information (Fig. S4), a high root-mean-square roughness results in a strong decay of the scattering intensity in the *q_z_* direction, while the scattering intensity extends to rather high values in the *q_y_* direction, especially at *q_z_* values near the Yoneda peak. In that case, it is possible to characterize the scattering from the rough substrate by analysing linecuts III and IV (*i.e.* high *q_z_*), and that by the rough sample holder by analysing linecuts I and IV [*i.e.* low *q_z_*, Fig. 4 ▸(*b*)].

Both *I*_DB_(*q_y_*, *q_z_*) and *I*_sur_(*q_y_*, *q_z_*) can be present simultaneously as background contributions in an experiment [Fig. 4 ▸(*c*)], especially when large beams are used at a low incident angle. It is also possible to address these two contributions separately by choosing appropriate linecuts. Once *I*_DB_(*q_y_*, *q_z_*) and *I*_sur_(*q_y_*, *q_z_*) are known from the measurements on the bare substrate, they can be included in the simulation of the 2D GISAXS pattern of the film sample, adjusting their relative contributions *A*_DB_ and *A*_sur_ and using the same linecuts as before, namely linecuts I, III, IV and V.

#### 2.2.3. Accounting for a finite beam size, beam divergence and wavelength spread

If the beam size is large compared with the pixel size of the detector, it may be necessary to include finite beam size effects in the simulation of the scattering of the bare substrate by applying a resolution function. This can be determined from a direct beam measurement. Neglecting the beam size, the scattering contribution of the substrate roughness may be particularly underestimated, since this contribution is mainly located around *q_y_* = 0. In many cases, it is sufficient to include only the effect of the lateral beam size. The vertical beam size can be neglected because, owing to the grazing-incidence geometry, the cross section of the sample with the beam in the normal direction is often smaller than the beam size itself, especially for small samples, which automatically gives a narrow resolution function (Pedersen *et al.*, 1990 ▸; Smilgies, 2009 ▸).

Additionally, a finite beam divergence and wavelength spread should be included in the simulation. Though these two effects are typically small for GISAXS experiments performed at dedicated synchrotron beamlines, it is necessary to include them for a quantitative analysis of the results (Smilgies, 2009 ▸). Typically, the two effects are included by applying a distribution function on the parameters for the incident angle and the wavelength.

To summarize, it is important to obtain as much information as possible on the substrate and the parasitic background scattering, such as the roughnesses of the substrate and the holder and the direct beam scattering, possibly by applying a resolution function. Ideally, a GISAXS measurement is carried out on the bare substrate under the same conditions (beamline, sample cell, sample size, incident angle) as the film sample. Using appropriate linecuts, the different background contributions can be identified and characterized, and the resulting structural parameters can be included in the simulation of the 2D GISAXS maps of the nanostructured film sample.

### 2.3. Step 2: adjusting the surface roughness, refractive index and thickness of the film

In the second step of the simulation, a homogeneous film with a finite thickness is placed on top of the substrate. The surface roughness of the film and correlated roughness effects are also considered in this step. There are two reasons to simulate a homogeneous film separately before including any nanostructures:

(i) The scattering contributions of the homogeneous film, which are mostly attributed to its surface roughness and interference effects due to its finite thickness, are strongest around *q_y_* = 0, but weak elsewhere. This makes it possible to isolate the parameters related to the homogeneous film in a separate fitting routine with a manageable number of fitting parameters.

(ii) In the DWBA, the homogeneous film is the basis of the dynamic scattering effects, while its nanostructure is treated as a perturbation and is calculated kinematically (Holý *et al.*, 1999 ▸). As such, it is advantageous to first obtain the parameters that are needed to establish the unperturbed scattering potential, before including any perturbations (nanostructures) in the simulations. This protocol also enables the calculation of the electric-field intensity within the film, which allows us to include waveguide effects in the simulations (Jiang *et al.*, 2011 ▸).

We note that the descriptions in this section regard films consisting of a single layer. Multilayers are conceptually similar but introduce many more parameters (several thicknesses, interface roughnesses and refractive indices), which can hardly be captured with GISAXS experiments alone. The 2D GISAXS patterns of such multilayer structures can, in principle, be simulated analogously. However, they should be pre-characterized by complementary measurements, such as XRR or ellipsometry, and this information should be included in the GISAXS simulations.

**Figure 5:**
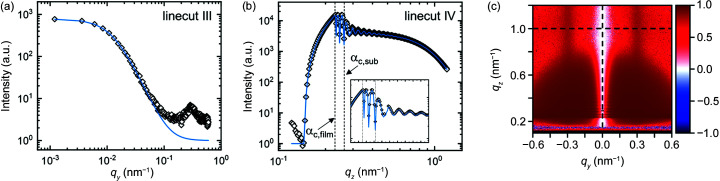
Linecuts (*a*) III at *q_z_* = 1.01 nm^−1^ and (*b*) IV of the simulated 2D GISAXS pattern of the representative nanostructured thin film with noise (step 4, open black symbols) and of a simulation including only the substrate with the homogeneous film on top (step 2, blue lines). The vertical dashed lines in (*b*) indicate the critical angles of the film and of the substrate. The inset in (*b*) shows a close-up of the Yoneda band region from 0.2 to 0.4 nm^−1^. (*c*) Residual plot given by (*I*_step4_ − *I*_step2_)/*I*_step4_. Horizontal and vertical dashed lines indicate the positions of linecuts III and IV, respectively.

The surface roughness of the film is obtained similarly to the substrate roughness (Section 2.2.1). Again, linecuts III and IV are considered, giving information about the root-mean-square roughness σ_rms,film_, the lateral correlation length of the roughness of the film surface ξ_film_ (linecut III) and the Hurst parameter *H*_film_, (linecut IV). The contributions of the supported, homogeneous film to these two linecuts are shown in Figs. 5 ▸(*a*) and 5 ▸(*b*), respectively. In these fits, it is important to keep the previously determined parameters of the substrate roughness constant. Furthermore, fits of linecuts III and IV should be performed simultaneously. For better results, it is advisable to measure the surface roughness of the film by AFM. The linecuts show that, up to *q_y_* = 0.1 nm^−1^, the amplitude and the shape of the decay in linecut III are mainly due to the scattering of the supported homogeneous film with the given roughnesses of the substrate and the film surface. The residual plot [Fig. 5 ▸(*c*)] demonstrates that the scattering contributions of the supported homogeneous film are dominant near *q_y_* = 0, but almost absent elsewhere, hence the strong deviations at *q_y_* ≳ 0.05 nm^−1^.

The Yoneda peak of the film in the model sample manifests itself as a peak at a lower *q_z_* value than that of the substrate, as seen by comparing the linecuts IV from the simulations of the bare substrate [Fig. 3 ▸(*b*)] and that of the homogeneous film on this substrate [Fig. 5 ▸(*b*)]. Together, they delimit a region of enhanced scattering intensity, which is often referred to as the Yoneda band. The critical angle can thus be obtained by reading off the *q_z_* position of the Yoneda peak in linecut IV [Fig. 5 ▸(*b*)] or, alternatively, linecut II. Between the two Yoneda peaks, waveguide oscillations are present, and their period and amplitude are well described by the simulation. To avoid confusion, it is advisable to calculate the refractive index of the film from its chemical composition and mass density.

A well defined film thickness results in intensity oscillations (called ‘fringes’) above the Yoneda peak of the substrate in linecut IV [Fig. 5 ▸(*b*)] and may be due to a correlated roughness of the substrate surface and the film surface or to dynamical scattering features (Müller-Buschbaum & Stamm, 1998 ▸; Holý *et al.*, 1999 ▸). The spacing of the fringes of these two film thickness effects differs by approximately a factor of two and, therefore, their origin must be carefully considered. Hence, it is advisable to obtain at least a rough estimate of the film thickness from complementary measurements, such as AFM, XRR, ellipsometry or spectral reflectance, to be able to distinguish between these two effects. In the case of correlated roughness between the substrate and the film surface, it may be necessary to include a finite vertical cross correlation length (ξ_⊥_) in the simulations (Holý & Baumbach, 1994 ▸).

Due to the limited *q_z_* range covered and the finite resolution of GISAXS measurements, film thicknesses that are too small or too large cannot be resolved. Moreover, a high surface roughness of the film smears the fringes, making it difficult to identify the characteristic minima or maxima. In these cases, the film thickness cannot be determined from the GISAXS data alone and must be obtained from complementary measurements.

To summarize, before adding structure to the film in the simulation, the surface roughness, the refractive index and the thickness of the film should be determined by simulating linecuts III and IV from a homogeneous film, possibly with the help of complementary measurements or calculations.

### 2.4. Step 3: adding structures to the film surface

Generally speaking, the nanostructure of the film can be separated into two contributions: the inner structure, which is buried within the film, and its surface structure. In step 3, the surface structure is added coherently to the simulation. Examples of surface structures are protrusions or nanoparticles decorating the surface of the film. There are several reasons why the surface structure can be treated separately from the inner structure.

(i) The scattering features of surface structures are often more prominent, since their contrast with the ambient environment is often higher than the contrast between the nanodomains within the film.

(ii) Scattering of the surface structure extends to exit angles below the critical angle of the film and is therefore recorded by the detector below the Yoneda band, whereas this is not the case for scattering from buried nanostructures.

(iii) The nanostructures at the surface are located on the 2D film surface which, in the case of ordered surface structures, gives rise to vertical scattering rods.

**Figure 6:**
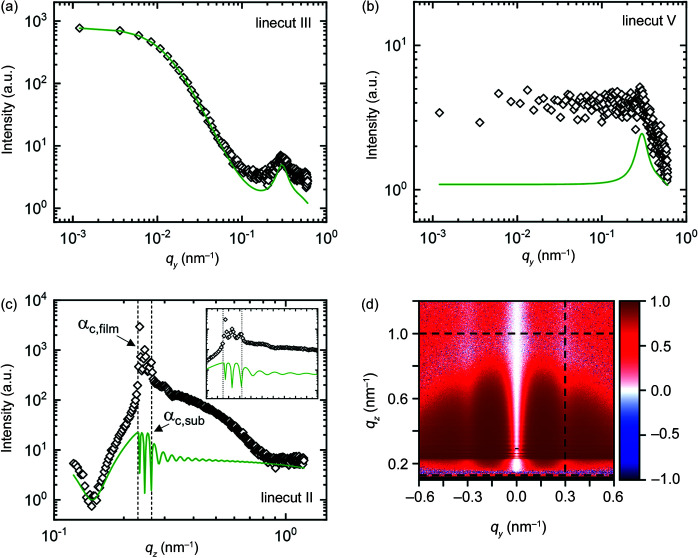
Linecuts (*a*) III at *q_z_* = 1.01 nm^−1^, (*b*) V at *q_z_* = 0.13 nm^−1^ and (*c*) II at *q_y_* = 0.29 nm^−1^ of the simulated 2D GISAXS pattern of the representative nanostructured thin film with noise (step 4, open black symbols) and of a simulation including only the substrate with the homogeneous film on top (step 3, green lines). The vertical dashed lines in (*c*) indicate the critical angles of the film and of the substrate. The inset in (*c*) shows a close-up of the Yoneda band region from 0.2 to 0.4 nm^−1^. (*d*) Residual plot given by (*I*_step4_ − *I*_step3_)/*I*_step4_. Horizontal and vertical dashed lines indicate the positions of linecuts III, V and II, respectively.

In the model sample, the ordered cylindrical nanopillars at the surface lead to scattering in the form of vertical scattering rods (Figs. 2 ▸ and S1 of the supporting information). Thus, linecuts III and V and linecut II at the *q_y_* position of the scattering rod (0.3 nm^−1^) are suitable choices to characterize the scattering from the surface structures separately. Linecut III contains a peak at the position of the scattering rod near *q_y_* = 0.3 nm^−1^, which can be fitted using the structure factor of a paracrystal combined with the form factor of the cylinders [Fig. 6 ▸(*a*)]. Linecut V also shows that the peak is present at this *q_y_* position, *i.e.* the vertical rod reaches down to *q_z_* below the Yoneda band [Fig. 6 ▸(*b*)]. The position of linecut II is chosen to be within the vertical rod and thus gives information about the form factor along the film normal [Fig. 6 ▸(*c*)]. When fitting linecut II, special focus should be on the *q_z_* range below the critical angle of the film, since in this region, the scattering is almost exclusively due to the surface structures and background scattering. For the model sample, a shoulder is present above the horizon at *q_z_* = 0.15 nm^−1^, which is due to the form factor of the cylindrical nanopillars. However, the high intensity at *q_z_* values above the Yoneda band cannot be reproduced with the surface structures alone [Fig. 6 ▸(*c*)]. Compared with step 2, the residual has improved in the regions *q_z_* > 0.8 nm^−1^ and *q_z_* < 0.2 nm^−1^ (*i.e.* at high *q_z_* values and below the Yoneda band) at all *q_y_* values as well as around *q_y_* = ±0.3 nm^−1^ at all *q_z_* values [*i.e.* along the vertical rods, Fig. 6 ▸(*d*)].

Again, it is advisable to complement the GISAXS measurements with measurements of the surface structure in real space, *e.g.* by AFM. This way, important insight on the shape and type of distribution of the surface structure as well as on the length scales can be gained, which limits the range of possible fit models and parameters.

### 2.5. Step 4: adding the inner film structure

After having accounted for the contributions from the substrate, the film itself and its surface structure in steps 1–3, only the contributions of the inner structure of the film remain to be included.

For these fits, linecuts I and II are primarily used. However, it may be helpful to include some of the other linecuts, if considerable scattering contributions are present in their respective regions, which were not fully described in the previous steps. For the model sample, these might be linecut III around the peak region (0.3 nm^−1^) and linecut V at *q_y_* values up to this value [Figs. S2(*c*) and S2(*e*) of the supporting information]. In step 4, the major challenge lies in the identification of suitable form and structure factors. If possible, complementary techniques such as cross-sectional scanning electron microscopy (SEM) should be used to solidify their choice and reduce the ambiguities of scattering models (Pauw, 2013 ▸).

**Figure 7:**
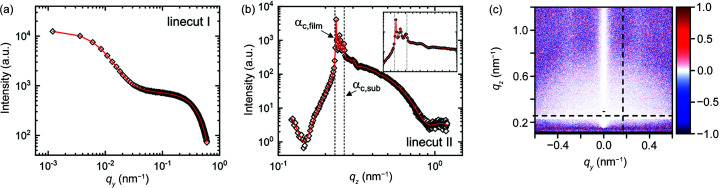
Linecuts (*a*) I at *q_z_* = 0.26 nm^−1^ and (*b*) II at *q_y_* = 0.17 nm^−1^ of the simulated 2D GISAXS pattern of the representative nanostructured thin film with noise (step 4, open black symbols) and of a simulation including only the substrate with the homogeneous film on top (step 4, red lines). The vertical dashed lines in (*b*) indicate the critical angles of the film and of the substrate. The inset in (*b*) shows a close-up of the Yoneda band region from 0.2 to 0.4 nm^−1^. (*c*) Residual plot given by (*I*_step4_ − *I*_step4_)/*I*_step4_. Horizontal and vertical dashed lines indicate the positions of linecuts I and II, respectively.

Linecuts I and II of the model sample from step 4 are shown in Figs. 7 ▸(*a*) and 7 ▸(*b*), respectively. Note, in step 4, the *q_y_* position of linecut II is chosen to be outside the vertical rod, which is different from step 3. The following observations can be made: (i) As seen above [Fig. 5 ▸(*a*)], the shoulder in linecut III at *q_y_* ≅ 0.01 nm^−1^ is fully described by the surface roughnesses of the substrate and the film. Thus, for the fit of the inner structure, only the shoulder at *q_y_* > 0.1 nm^−1^ is relevant. Without this information, the former shoulder may falsely be attributed to a large-scale structure scattering at low *q_y_* values. (ii) In linecut II, strong dynamical scattering features are present in the region of the Yoneda band. This region is excluded from the fit in many analysis methods, since it is rather complex and would introduce too many fitting parameters. However, because steps 1–2 have independently addressed these parameters, they can be included and kept fixed. These observations highlight the advantage of the stepwise approach. The entire *q_z_* range, especially the shoulder above the Yoneda band, is well described [Figs. 7 ▸(*b*) and 7 ▸(*c*)]. The region around the peak in linecut III is now well described [Fig. S2(*c*)].

Finally, it should be verified that the fit of the surface structures in step 3 was not significantly changed by the addition of the inner structures in step 4. Since the scattering is coherent, dependencies may arise which were not previously included in the fit in step 3. It may therefore be necessary to revisit the structural parameters of the surface structures in step 4 and allow them to vary during the fit.

### 2.6. Summary of the stepwise simulation strategy

A summary of the four steps of the strategy along with suggestions for complementary measurements and the addressed parameters is given in Table 1 ▸. In step 1, the investigation of a bare substrate yields the parameters of the substrate surface roughness and refractive index as well as a set of parameters describing parasitic background contributions, if present. Step 2 yields the parameters of the film surface roughness and refractive index as well as the film thickness and roughness correlations. Together, steps 1 and 2 yield parameters that are relevant for the treatment of dynamical scattering effects of the film. Steps 3 and 4 yield sets of parameters describing the surface structure and inner structure, respectively. We note that nanostructured multilayers can, in principle, be treated in the same way, ideally with additional information from other sources.

## 3. Examples

To demonstrate the applicability of the presented strategy to the modelling of GISAXS data from a real nanostructured film and to highlight its benefits, we present the measured data from one of our recent investigations of thin films from multiblock polymers (Jung *et al.*, 2021 ▸) and show the stepwise model fitting.

### 3.1. Film with cylindrical morphology and structured surface

**Figure 8:**
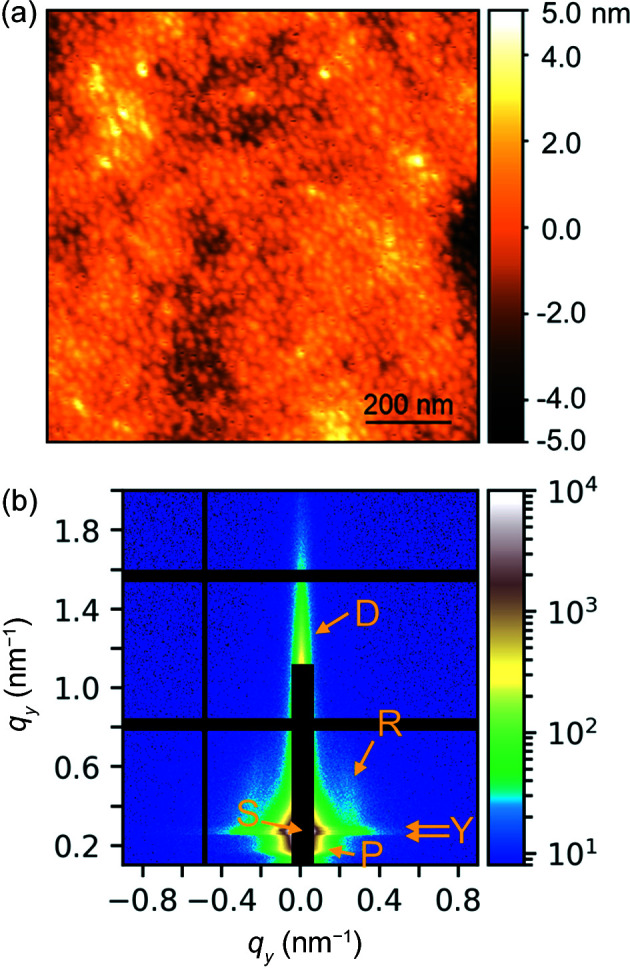
Experimental data from a nanostructured block copolymer film. (*a*) AFM height image. The scale bar and the height scale are given. (*b*) Measured 2D GISAXS pattern. The logarithmic intensity scale is given on the right. The black stripes are detector gaps. Arrows indicate the calculated position of the specularly reflected beam (S), specularly diffuse scattering (D), the critical angles calculated for the substrate (α_c,sub_) and the film (α_c,film_) delimiting the Yoneda band Y [for details see Jung *et al.* (2021 ▸)], vertical scattering rods (R), and a scattering peak below the Yoneda band (P).

The sample under consideration is a thin film from a doubly pH-responsive pentablock terpolymer of type A-*b*-B-*b*-C-*b*-B-*b*-A (Jung *et al.*, 2021 ▸). The B and C blocks are poly[2-(dimethylamino)ethyl methacrylate] (PDMAEMA) and poly(2-vinyl­pyridine) (P2VP), respectively, which are both cationic polyelectrolytes having different p*K*_a_ values, namely 7.5 and 5.0, respectively. The A blocks are short poly(methyl methacrylate) (PMMA) blocks. Thus, the pentablock terpolymer has different charge states depending on the pH value. The aim of the study was to investigate the structure in thin films depending on the charge state. Here, we present data from a film that was spin-coated from aqueous solution at pH 7.1 onto an Si substrate. It was 267 nm thick, as measured by spectral reflectance, and 1.5 × 1.5 cm large. For details of the film preparation, we refer to our previous publication. The AFM height image of a similar film prepared at pH 7.2 showed circular protrusions with radii of ∼6 nm, distances of ∼29 nm and heights of ∼0.7 nm [Fig. 8 ▸(*a*)]. In a number of images, we also observed large protrusions, especially at pH 8.3 (Jung *et al.*, 2021 ▸).

GISAXS experiments were carried out at the Austrian SAXS beamline at the Elettra Sincrotrone Trieste (Amenitsch *et al.*, 1998 ▸) under standard conditions; for details see Jung *et al.* (2021 ▸). The wavelength was 0.155 nm, the beam size 1.0 × 0.15 mm (H × V) and the incident angle 0.195°. The detector, a PILATUS3 1M (981 × 1042 pixels with a pixel size of 172 µm), was placed at a sample-to-detector distance of 1951 mm. The sample holder was made from aluminium, and the length of the support in the beam direction was 30 mm; thus, nominally, the footprint of the beam was larger than the sample for incident angles below 0.573° and larger than the sample holder for incident angles below 0.286°. Therefore, background contributions from the sample holder can be expected to be present in 2D GISAXS patterns.

The 2D GISAXS pattern of the supported thin polymer film is shown in Fig. 8 ▸(*b*). The specularly reflected beam (S) is located at *q_z_* = 0.27 nm^−1^ and the calculated Yoneda band (Y) between *q_z_* = 0.25 nm^−1^ and *q_z_* = 0.30 nm^−1^. Moreover, the pattern features a vertical streak along *q_y_* = 0 (D), vertical scattering rods (R) at *q_y_* ≃ 0.3 nm^−1^ and a scattering peak below the Yoneda band (P). Traditionally, such a pattern would be attributed to the presence of standing cylinders in the film. The lateral repeat distance would be determined from the peaks in a horizontal linecut at the *q_z_* value of the critical angle of the film, and the degree of order from the width of the reflection and the presence and amplitude of higher-order reflections. However, typically, the detailed shape of the reflection, its length along *q_z_* as well as the meaning of the streak, the diffuse scattering extending to high *q* values, and the parasitic scattering could not be easily revealed by such a simplified approach. Such problems are inherent to weakly ordered soft-matter films. As an alternative, one of the software packages named above could be used to construct a model consisting of standing cylinders that are correlated via a paracrystalline structure factor. However, in both approaches, certain features could be misinterpreted, for instance, when background contributions are not carefully taken into account. Here, we present how our strategy can be used to quantitatively describe the entire 2D pattern by including not only the inner film structure but also its surface structure and background contributions into the model. As a software package, *BornAgain* was used (version 1.19.0; Burle *et al.*, 2018 ▸; Pospelov *et al.*, 2020 ▸).

**Figure 9:**
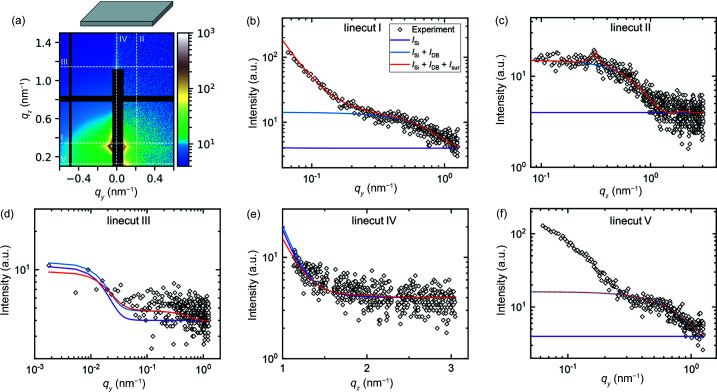
(*a*) Experimental (*q_y_* > 0) and simulated (*q_y_* < 0) 2D GISAXS pattern of a bare Si substrate at an incident angle of 0.22°. Dashed white lines indicate the positions of linecuts I–V. (*b*)–(*f*) Linecuts I–V of experiment (open symbols) and simulation (solid lines). The purple line is the best fit using a single substrate with low roughness. The blue line is the best fit including an incoherent addition of direct beam scattering of a spherical object. The red line is the best fit using the simulation setup as described in the text. In all three cases, a constant background is included. 2D patterns and residual plots of each case are shown in Fig. S7. In (*f*), the blue and red lines overlap.

In order to characterize the substrate and to identify background contributions in the 2D pattern of the supported polymer film, a complementary measurement of a bare substrate was performed under the same conditions. The simulation describing the background is chosen to be the incoherent sum of a substrate with low surface roughness (*I*_Si_), direct beam scattering of a spherical object (*I*_DB_) and a substrate with high surface roughness (*I*_sur_) as well as a constant background [following equation (4)]. The parameters of the simulation are summarized in Table S2 of the supporting information. The 2D GISAXS pattern is shown in Fig. 9 ▸(*a*). The linecuts along with best fits of *I*_Si_, *I*_Si_ + *I*_DB_ and *I*_Si_ + *I*_DB_ + *I*_sur_ are shown in Figs. 9 ▸(*b*)–9 ▸(*f*). *I*_Si_ gives a reasonable description of the specularly diffusive scattering near *q_y_* = 0 (linecuts III and IV), but captures neither the features near the Yoneda band (linecuts I and II) nor the scattering below the horizon in linecut V [purple line in Figs. 9 ▸(*b*)–9 ▸(*f*)]. The addition of *I*_DB_ describes the scattering below the horizon (linecut V) and explains the shoulders in linecuts I and II [blue line in Figs. 9 ▸(*b*)–9 ▸(*f*)]. Finally, the addition of *I*_sur_ describes the decay at low *q_y_* in linecut I and the Yoneda Peak at *q_z_* ≃ 0.3 nm^−1^ in linecut II [red line in Fig. 9 ▸(*b*)–9 ▸(*f*)]. The shoulder at low *q_y_* in linecut V is attributed to direct beam scattering of larger particles but is neglected in the model since it does not have significant scattering contributions above the horizon.

**Figure 10:**
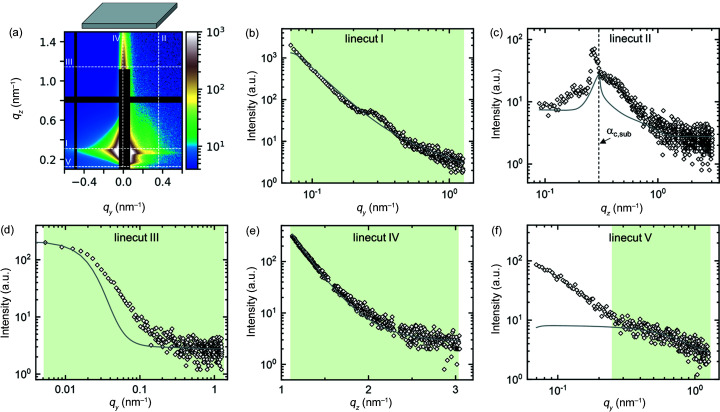
(*a*) Experimental (*q_y_* > 0) and simulated (*q_y_* < 0) 2D GISAXS patterns of the example film at an incident angle of 0.20° at step 1. Dashed white lines indicate the positions of linecuts I–V. The residual plot is shown in Fig. S8(*a*). (*b*)–(*f*) Linecuts I–V of experiment (open symbols) and simulation (solid lines). Green areas indicate the range of each linecut included in the fit.

Using the same simulation setup and parameters of the bare substrate and of the background contributions from Fig. 9 ▸ and Table S2, a best fit to the GISAXS data of the supported film is performed (step 1 of the stepwise strategy). In the fit, only the amplitudes of the different contributions are allowed to vary [see equation (4)]. The resulting 2D scattering image and linecuts I–V are shown in Fig. 10 ▸ and the parameters are given in Table S3. Only linecut IV fits reasonably well to the experimental data. The shoulder of the experimental data in linecut III is broader than the simulation of the bare substrate, indicating that the lateral correlation length of the film surface is smaller than that of the substrate (see Fig. S5). In linecut I, a peak is present at *q_y_* ≃ 0.3 nm^−1^, which indicates the presence of a nanostructure. This peak is associated with the scattering rods identified in Fig. 7 ▸. In linecut II, the Yoneda peak of the substrate is visible at *q_z_* ≃ 0.3 nm^−1^ and matches with the upper end of the Yoneda band of the experimental data. An additional peak is present at *q_z_* ≃ 0.2 nm^−1^, which is associated with the additional scattering peak below the Yoneda band in Fig. 7 ▸, but cannot be described by the scattering of the substrate or the background contributions. In linecut V, no additional background contributions other than those identified in the measurement of the bare substrate in Fig. 9 ▸ are observed.

**Figure 11:**
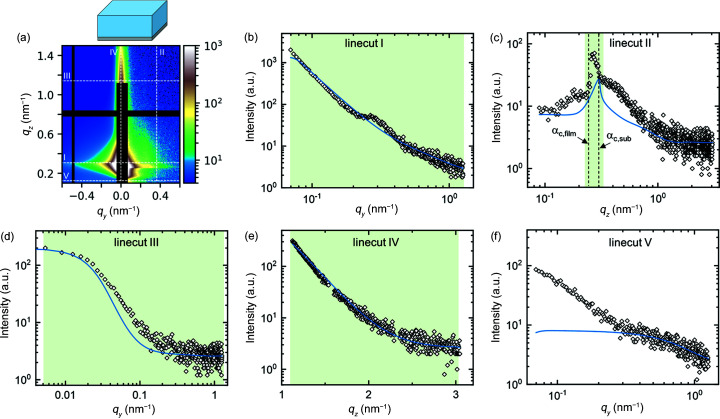
(*a*) Experimental (*q_y_* > 0) and simulated (*q_y_* < 0) 2D GISAXS patterns of the example film at an incident angle of 0.20° at step 2. Dashed white lines indicate the positions of linecuts I–V. The residual plot is shown in Fig. S8(*b*). (*b*)–(*f*) Linecuts I–V of experiment (open symbols) and simulation (solid lines). Green areas indicate the range of each linecut included in the fit.

In step 2, a homogeneous film is added to the simulation. The film thickness is kept fixed at 267 nm, *i.e.* at the value measured by spectral reflectance. The characteristic fringes in the experimental data are too weak to allow a fit of the film thickness. No vertical correlation is assumed. The refractive index of the film was estimated from its chemical composition and was adjusted by hand to fit the critical angle of the film observed in linecut II of the experimental data. The best fit, in which only the amplitudes of the different contributions and the root-mean-square roughness, lateral correlation length and Hurst parameter of the films surface roughness are allowed to vary, is shown in Fig. 11 ▸. The resulting parameters are given in Table S3. With the addition of the homogeneous film to the simulation, the fit of linecut IV has improved and the shoulder in linecut III has broadened. However, the scattering features in linecuts I and II are not yet fully described.

**Figure 12:**
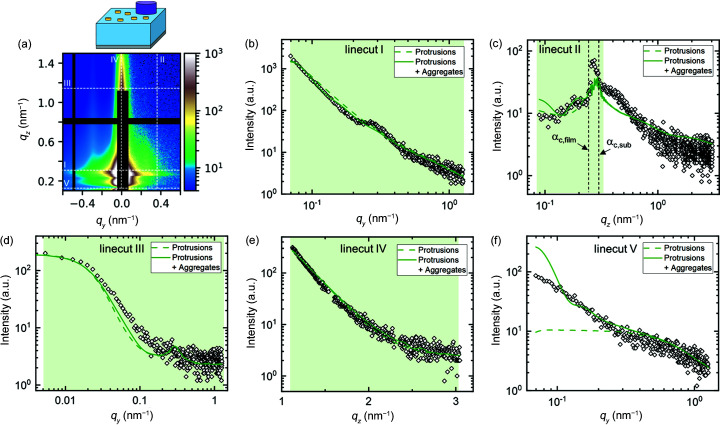
(*a*) Experimental (*q_y_* > 0) and simulated (*q_y_* < 0) 2D GISAXS patterns of the example film at an incident angle of 0.20° at step 3. Dashed white lines indicate the positions of linecuts I–V. The residual plot is shown in Fig. S8(*c*). (*b*)–(*f*) Linecuts I–V of experiment (open symbols) and simulation (solid lines: protrusions only; dashed lines: protrusions and aggregates). Green areas indicate the range of each linecut included in the fit.

In step 3, a surface structure is added to the simulation. The protrusions observed in the AFM image in Fig. 8 ▸(*a*) are modelled by standing cylinders which are correlated like a radial paracrystal. In the fit, the values of protrusion radius, height and spacing are set to 6, 1 and 20 nm, respectively, which are close to the values observed by AFM. The best fit is shown as dashed lines in Fig. 12 ▸. The scattering contribution of the protrusions is a peak in linecut III at *q_y_* ≃ 0.3 nm^−1^. A full fit of the structural parameters of the protrusions is not possible since their contribution is very weak. In fact, without the evidence from the AFM measurements, the GISAXS data alone do not indicate their presence. It is, however, possible to verify the order of magnitude of the structural parameters. The addition of the protrusions does not reproduce the scattering peak below the Yoneda band in linecut II. A good fit in this region was achieved by adding uncorrelated cylindrical aggregates with a radius and height of ∼30 nm to the simulation (solid lines in Fig. 12 ▸). They are absent in the AFM image in Fig. 7 ▸, but they were observed in AFM images of similar films (Jung *et al.*, 2021 ▸). The simulated aggregates also produce significant scattering at low *q_y_* in linecut V, which is an artefact of the simulation (direct beam scattering below the sample horizon, which is not observable in the experiment).

**Figure 13:**
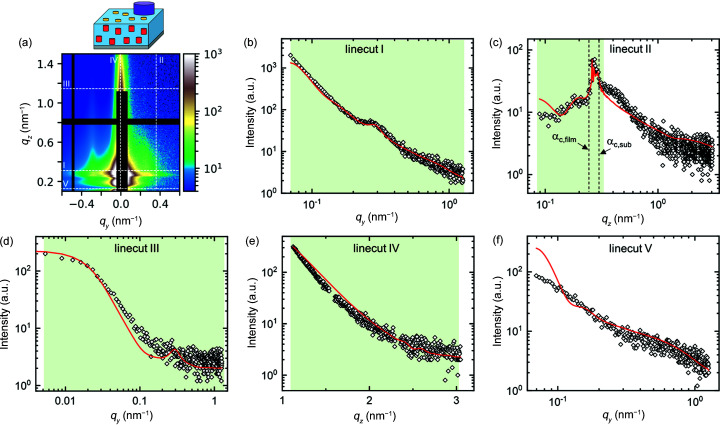
(*a*) Experimental (*q_y_* > 0) and simulated (*q_y_* < 0) 2D GISAXS patterns of the example film at an incident angle of 0.20° at step 4. Dashed white lines indicate the positions of linecuts I–V. The residual plot is shown in Fig. S8(*d*). (*b*)–(*f*) Linecuts I–V of experiment (open symbols) and simulation (solid lines). Green areas indicate the range of each linecut included in the fit.

Finally, in step 4, the inner film structure is added to the simulation. Indications of the presence of an inner structure are that, in step 3, the peak in linecut I and the Yoneda band in linecut II could not be described satisfactorily. The inner structure is modelled by standing cylinders which are randomly distributed in the film normal direction but are correlated in the film plane like a radial paracrystal. The height of the cylinders is manually set to 10 nm. The best fit, in which the amplitudes of all contributions as well as the radius and spacing of the inner structure are allowed to vary, is shown in Fig. 13 ▸. The peak in linecut I is now well described, and the intensity in the Yoneda band matches the experimental intensity. Moreover, the shoulder in linecut II above the Yoneda band is fitted slightly better compared with step 3. The remaining mismatch between the experiment and the simulation is due to the fact that the cylinders inside the film are not perfectly straight, as indicated by the slight inward bend of the Bragg rods, and this bend is not included in the simulation.

This example highlights the importance of complementary measurements to provide input for the simulation model at the different steps. In particular, AFM images of the film surface allowed us to identify the contributions of the surface structure to the GISAXS data and enable a quantitative fit of the inner structure parameters. The results of the structural investigation provide valuable insights into the complex film structures of multiblock polymers with charged blocks. Only quantitative GISAXS simulations allow detailed information to be obtained from these weakly scattering and weakly ordered systems on multiple length scales, following the strategy outlined in this work, and capturing all relevant features of the 2D GISAXS patterns.

## 4. Conclusions

A powerful and robust strategy to simulate and fit 2D GISAXS patterns of nanostructured thin films is introduced. The strategy is based on four steps which focus on the contributions of the bare substrate (step 1), the homogeneous film (step 2), the surface structure (step 3) and the inner structure (step 4), respectively. The stepwise approach allows us to separately determine a subset of structural parameters, which reduces the complexity and the computational demand of the simulations. It is also a guideline to follow when analysing GISAXS data of thin films by simulations. Furthermore, the stepwise approach can be translated and applied to GISANS data (Müller-Buschbaum, Gutmann *et al.*, 2004 ▸; Korolkov *et al.*, 2012 ▸; Müller-Buschbaum, 2013 ▸; Nouhi *et al.*, 2017 ▸).

While the strategy can be applied to single GISAXS measurement of thin films, it is highly advisable to complement the GISAXS data with a few additional measurements to reduce the ambiguities in the interpretation of the data. The most useful additional measurements that are identified are GISAXS measurements of the bare substrate and AFM measurements of the thin film. These measurements come at little additional cost but greatly improve the quality of the simulations.

The strategy presented allows access to information which is rarely extracted from GISAXS data, such as the surface roughness of the film or the distinction between surface structures and inner structures. It allows fitting of vertical linecuts, which is difficult due to distortion and dynamical scattering effects. For that purpose, steps 1 and 2 of the strategy focus on the determination of those parameters which are needed to include the dynamical scattering effects. Quantitative information about the size, shape and distribution of the surface and inner structure of the film is obtained in steps 3 and 4, respectively. Since other scattering contributions are addressed in steps 1 and 2, it is now possible to identify suitable form and structure factors describing these structures. In summary, the strategy is well suited to reduce the complexity of GISAXS simulations and makes them more accessible for data analysis.

Looking ahead, it is possible to imagine software that provides a library of sample setups which can be used for simulations, as in common software used for fitting transmission small-angle scattering data (Förster *et al.*, 2010 ▸; Breßler *et al.*, 2015 ▸; Doucet *et al.*, 2021 ▸). For each step of the strategy, a selection of setups would be available to build up the simulation stepwise. For example, for step 1, commonly used substrates would be available. For step 2, films with different roughness profiles might be selected. In step 3, different predefined surface structures, such as spherical nanoparticles or cylindrical protrusions, might be used as templates. Similarly, for step 4, different inner film structures such as randomly distributed spheres, stacked lamellae or standing cylinders would be possible choices. Instead of making all those choices at once, at each step of the protocol, an informed choice for a part of the simulation would be made. For a series of measurements, *e.g.* for time-resolved experiments, the four fitting steps can be automatized after the initial choice of the model setup (Santoro & Yu, 2017 ▸; Wang *et al.*, 2018 ▸). Moreover, the residuals may serve as an input for artificial intelligence for the classification of nanostructures (Liu *et al.*, 2019 ▸).

Since at each step only a few linecuts rather than the full 2D GISAXS pattern are used in the fitting protocol, the stepwise approach only needs a short computation time. Furthermore, it is not necessary to save the full scattering pattern and therefore the data storage load is reduced. It might become possible to obtain structural representations of the sample in quasi-real time, for example, during beam time.

## Supplementary Material

Supporting figures and tables. DOI: 10.1107/S1600576723006520/ge5137sup1.pdf

## Figures and Tables

**Table 1:** Summary of steps and procedures in the order of application Sets of parameters are indicated by **P**. The purpose of the linecuts is discussed in Section 2.1 and their locations are shown in Fig. 2 ▸.

Step	Procedure	Complementary measurements	Parameters	Linecuts
1	Determine the surface roughness of the substrate	GISAXS of bare substrate	σ_rms,sub_, ξ_sub_, *H*_sub_	III, IV
	Determine the refractive index of the substrate		δ_sub_, β_sub_	IV
	Identify additional background contributions from the holder		**P**_background_	I, III, V
2	Determine the surface roughness of the film	AFM, XRR, ellipsometry	σ_rms,film_, ξ_film_, *H*_film_	III, IV
	Determine the refractive index of the film		δ_film_, β_film_	II, IV
	Determine the film thickness		*t*	II, IV
	Check for correlated or uncorrelated surface roughness of the film		ξ_⊥_	II, IV
3	Check for the existence of a nanostructure at the film surface	AFM, SEM	**P**_surface_	I, II, III
4	Check for the existence of nanostructures inside the film	Cross-sectional SEM	**P**_inner_	I, II
