# Supplementary material for: Strategy to simulate and fit 2D grazing-incidence small-angle X-ray scattering patterns of nanostructured thin films
Source: J Appl Crystallogr. 2023 Aug 16;56(Pt 5):1330–47. doi: 10.1107/S1600576723006520 (PMC10543672; doi:10.1107/S1600576723006520)
Supplement: Supplementary file 1 [file j-56-01330-sup1.pdf]

JOURNAL OF
APPLIED
CRYSTALLOGRAPHY

Volume 56 (2023)

Supporting information for article:

Strategy to simulate and fit 2D GISAXS patterns of nanostructured thin films

Florian A. Jung and Christine M. Papadakis

S1. Parameters used for the simulation of the model film

A model sample was constructed as follows (Figure S1a, all structural parameters are given in Table S1): A film with a thickness $t = 100$ nm and a surface roughness $\sigma_{rms,film} = 1.0$ nm is placed on top of an infinitely thick substrate, that has a surface roughness $\sigma_{rms,sub} = 0.1$ nm. For the real and imaginary parts of the refractive index of the substrate, δ_{sub} and β_{sub}, values similar to that of Si are used. The film surface is in vacuum. Cylindrical nanopillars of radius 5 nm and height 2 nm are placed on top of the film surface and are distributed according to a radial paracrystal. The nanopillars are assigned δ_{pil} and β_{pil} values typical for polymers. Randomly distributed nanospheres of radius 5 nm with a Gaussian size distribution having a width of 0.5 nm are buried inside the film. Their δ and β values are chosen different from the film to ensure a significant scattering contrast.

The beam is chosen to impinge on the surface of the film under an incident angle of 0.2°, which, at the wavelength given (0.15 nm), is slightly higher than the critical angles of the film ($\alpha_{c,film} = 0.115°$) and of the substrate ($\alpha_{c,sub} = 0.162°$). Thus, the X-ray beam penetrates the film fully and is partially reflected by the film-substrate interface. This angle has often been used, because it results in higher scattering intensities than higher incident angles, facilitating time-resolved measurements. The 2D GISAXS pattern of the model sample was simulated using BornAgain v1.19.0 (Figure S1b). At this, random noise is introduced by transforming each pixel $I(q)$ according to a Gaussian distribution with a standard deviation of $\sigma_{noise}(q) = \gamma_{noise} I(q)^{1/2}$, where $\gamma_{noise} = 0.5$ is a scale factor. For the sake of clarity, no additional parasitic scattering background was included in the model.

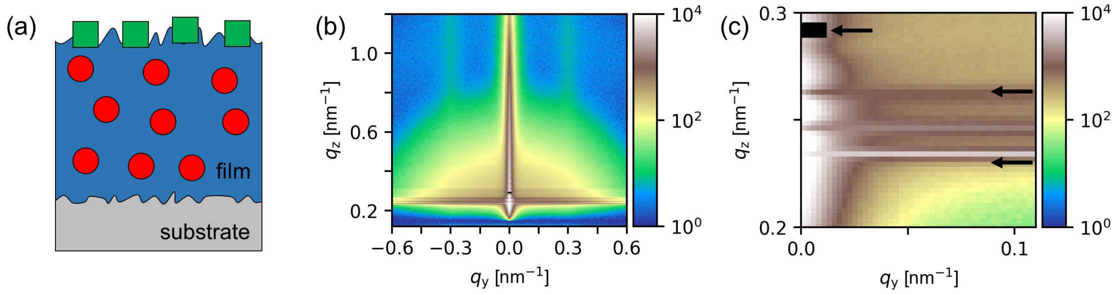

Figure S1 (a) Sketch of the model sample. (b) Simulated 2D GISAXS pattern of the model sample characterized by the parameters given in Table S1. (c) Close-up of (b) in the Yoneda band region. The arrows indicate (from top to bottom) the calculated positions of the reflected beam (covered by a mask) and the critical angles of the substrate and the film.

Table S1 Parameters used in the simulation of the model film

Instrumental parameters	Wavelength, λ	0.15 nm
	Sample-detector distance, *sdd*	3000 mm
	Detector dimension	500 × 500 pixels
	Pixel size, p_s	172 µm
	Direct beam position	250, 0
	Incident angle, α_i	0.2°
	Beam intensity, I_0	10^{14}
Substrate → addressed in step 1	Dispersion coefficient, δ_{sub}	4×10^{-6}
	Absorption coefficient, β_{sub}	6×10^{-8}
	Root-mean-square roughness, $\sigma_{rms,sub}$	0.1 nm
	Lateral correlation length, ξ_{sub}	200 nm
	Hurst parameter, H_{sub}	1.0
Film → addressed in step 2	Dispersion coefficient, δ_{film}	2×10^{-6}
	Absorption coefficient, β_{film}	3×10^{-9}
	Root-mean-square roughness, $\sigma_{rms,film}$	1.0 nm
	Lateral correlation length, ξ_{film}	100 nm
	Hurst parameter, H_{film}	0.5
	Film thickness, *t*	100 nm
	Vertical cross-correlation, $\xi_{\perp}$	0 nm (uncorrelated)
Cylindrical nanopillars at film surface → addressed in step 3	Radius, r_{cyl}	5 nm
	Height, h_{cyl}	2 nm
	Average spacing, d_{cyl} assuming a radial paracrystal	20 nm
	Width of Gaussian distribution of the paracrystal, ω_{cyl}	5 nm
Spheres inside film → addressed in step 4	Radius, r_{sphere}	5 nm
	Width of Gaussian distribution of sphere radius, σ_{sphere}	0.5 nm
Other	Constant background, I_{cbg}	1
	Noise scale factor, γ_{noise}	0.5

The 2D GISAXS pattern of the model sample features high intensity in the region q_z = 0.2-0.3 nm^{-1}, which is extended along the q_y axis and comprises a few intensity oscillations along q_z. This is the so-called Yoneda band, i.e. the region between the critical angles of the substrate ($\alpha_{c,sub}$ = 0.162°) and the polymer film ($\alpha_{c,film}$ = 0.115°), i.e. q_z = 0.265 and 1.230 nm^{-1}. The reflected beam is at q_z = 0.292 nm^{-1} and q_y = 0 and is masked in the simulation. A high-intensity streak centred at q_y = 0, that extends

along q_z, is observed and features intensity oscillations as well. It is due to scattering and reflections from the film/vacuum and the film/substrate interfaces, and its width in q_y is related to their roughnesses. Such streaks, albeit of weaker intensity, are also present at $q_y = \pm 0.3$ nm^{-1}, indicating a certain periodicity in the film plane. Generally speaking, the length of the streak is related to the height of the features which are at its origin. Finally, a halo of scattering extends around the reflected beam, which seems to contain information about the structure within the film. At q_z values below the critical angle of the film, the halo-like scattering quickly decays, while significant scattering of the vertical streaks remains.

These features are reflected in the 5 linecuts, that are described in the main text (see Figure 1). They are shown in Figure S2 along with the contributions calculated in the 4 steps. Linecut I is the intensity profile along q_y in the Yoneda band. Linecut II describes the enhanced intensity in the Yoneda band as well as the intensity oscillations within. Linecut III shows both, the intensity decay around $q_y = 0$ and the position of the streaks at $q_y = \pm 0.3$ nm^{-1}. Linecut IV is the intensity profile along q_z at $q_y = 0$ and contains the same oscillations as linecut II. In addition, it features intensity oscillations above the Yoneda band. Linecut V is constant, has a weak maximum at $q_y = 0.3$ nm^{-1}, presumably due to the streaks due to the protrusions, and a strong decay above.

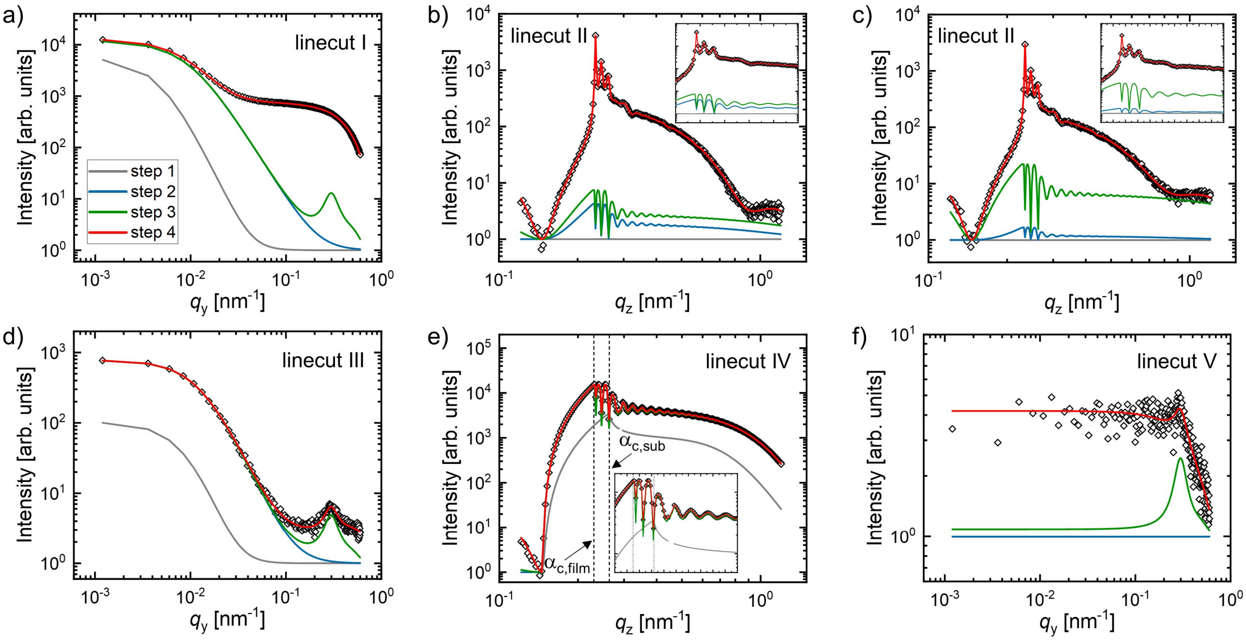

Figure S2 Linecuts I at $q_z = 0.26$ nm^{-1} (a), II at $q_y = 0.17$ nm^{-1} (b), II at $q_y = 0.29$ nm^{-1} (c) III at $q_z = 1.01$ nm^{-1} (d), IV (e) and V at $q_z = 0.13$ nm^{-1} (f) of the 2D GISAXS pattern of the model sample (open symbols) and of the fits in steps 1 (grey lines), step 2 (blue lines), step 3 (green lines) and step 4 (red lines). Insets in (b), (c) and (e) are close-ups of the Yoneda band region between 0.2 and 0.4 nm^{-1}.

S2. Linecuts of background contributions

Figure S3 shows linecuts I, III, IV and V of I_{sample}, I_{DB}, I_{sur} and their sum as given in Figure 4 in the main text.

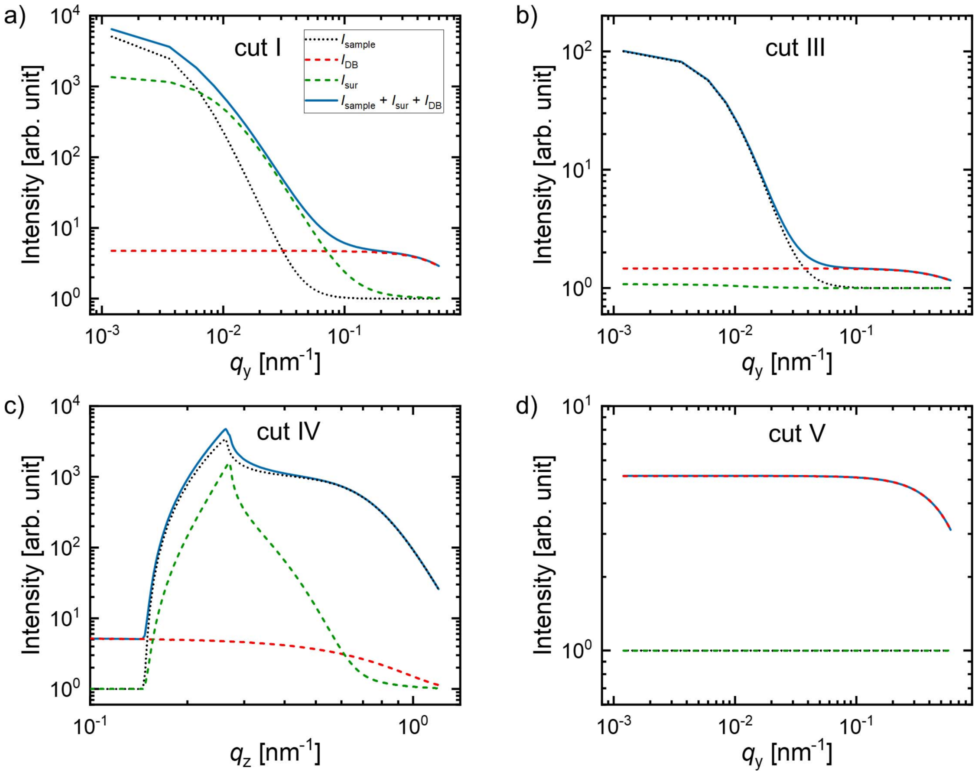

Figure S3 Linecuts I (a), III (b), IV (c) and V (d) of GISAXS patterns of the sample (black dotted line) and the background contributions I_{DB} (red dashed line) and I_{sur} (green dashed line) given in Figure 4. The sum of the three linecuts is shown as a blue full line.

S3. Simulations of substrates for different roughness parameters

Simulations of bare substrates are performed to illustrate the dependence of the scattering patterns on the surface roughness parameters. At this, the parameters in Table S1 were used (excluding the film and its surface and inner structure) and varying either the root-mean-square roughness, $\sigma_{rms,sub}$, the Hurst parameter H_{sub} or the lateral correlation length ξ_{sub}.

The effect of $\sigma_{rms,sub}$ on the 2D GISAXS patterns of the bare substrate and the corresponding linecuts III and IV is shown for for ξ_{sub} = 200 nm and H_{sub} = 1.0 in Figure S4. With increasing $\sigma_{rms,sub}$, the intensity of the vertical scattering streak is reduced at high q_z values (Figure S4c and e). At the same time, an overall increase of intensity is observed. The shape of the horizontal linecut III is, apart from the increase in intensity, unaffected by a change of σ_{rms} (Figure S4b and d).

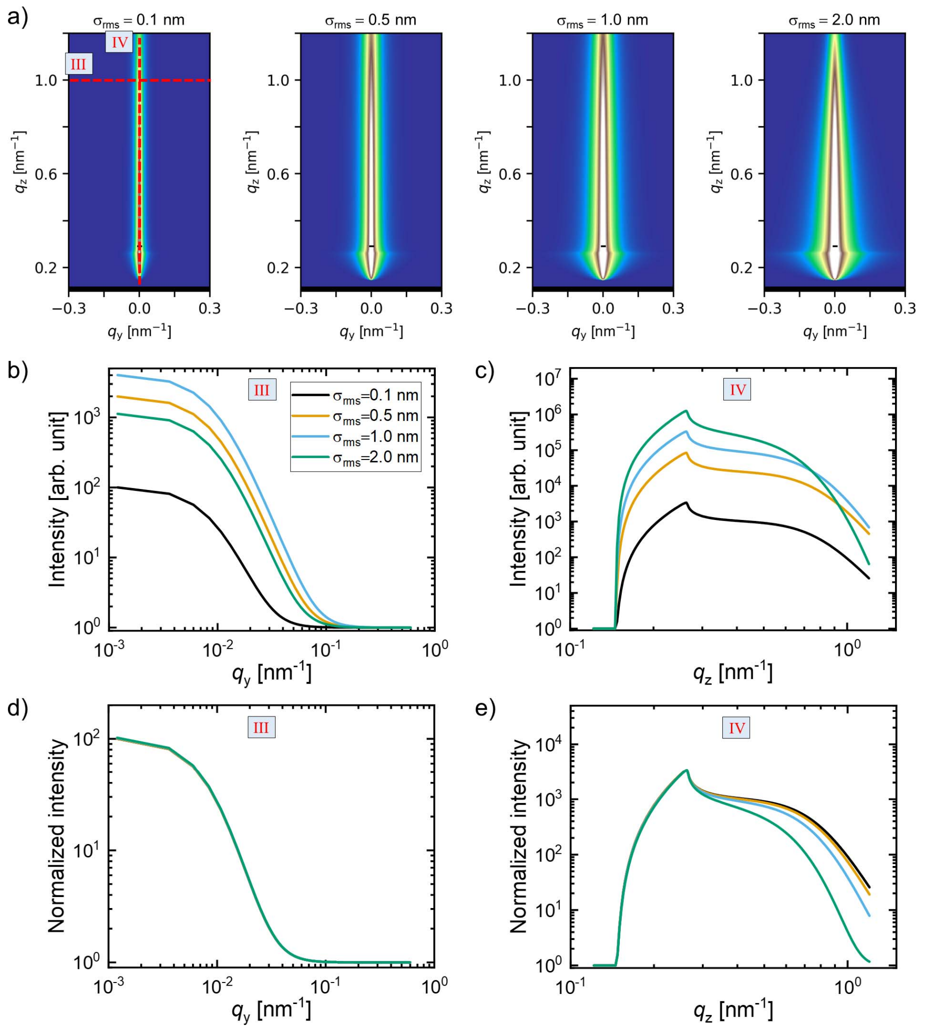

Figure S4 (a) Simulated 2D GISAXS patterns of bare substrates having root-mean-square roughnesses, $\sigma_{\rm rms}$, of 0.1 nm, 0.5 nm, 1.0 nm and 2.0 nm (left to right). All other parameters in the simulations are the same as in Table S1, i.e., $H = 1.0$ and $\xi = 200$ nm. (b, c) Linecuts III (b) and IV (c) of the patterns in (a). (d, e) Same linecuts as in (b) and (c), but normalized to the highest intensity of the cuts at $\sigma_{\rm rms} = 0.1$ nm. Note that the normalization factors in (d) and (e) are different to ensure maximum overlap of the respective curves.

The effect of $\xi_{\rm sub}$ on the 2D GISAXS patterns of the bare substrate and the corresponding linecuts III and IV is shown for $\sigma_{\rm rms,sub} = 0.1$ nm and $H_{\rm sub} = 1.0$ in Figure S5. With decreasing $\xi_{\rm sub}$, the vertical streak broadens along $q_{\rm y}$, and its overall intensity decreases. This goes along with a shift of the onset of the intensity decay in the horizontal linecut III towards larger $q_{\rm y}$ (Figure S5b and d). The vertical linecut features a plateau which extends towards larger $q_{\rm z}$ values, when ξ is decreased.

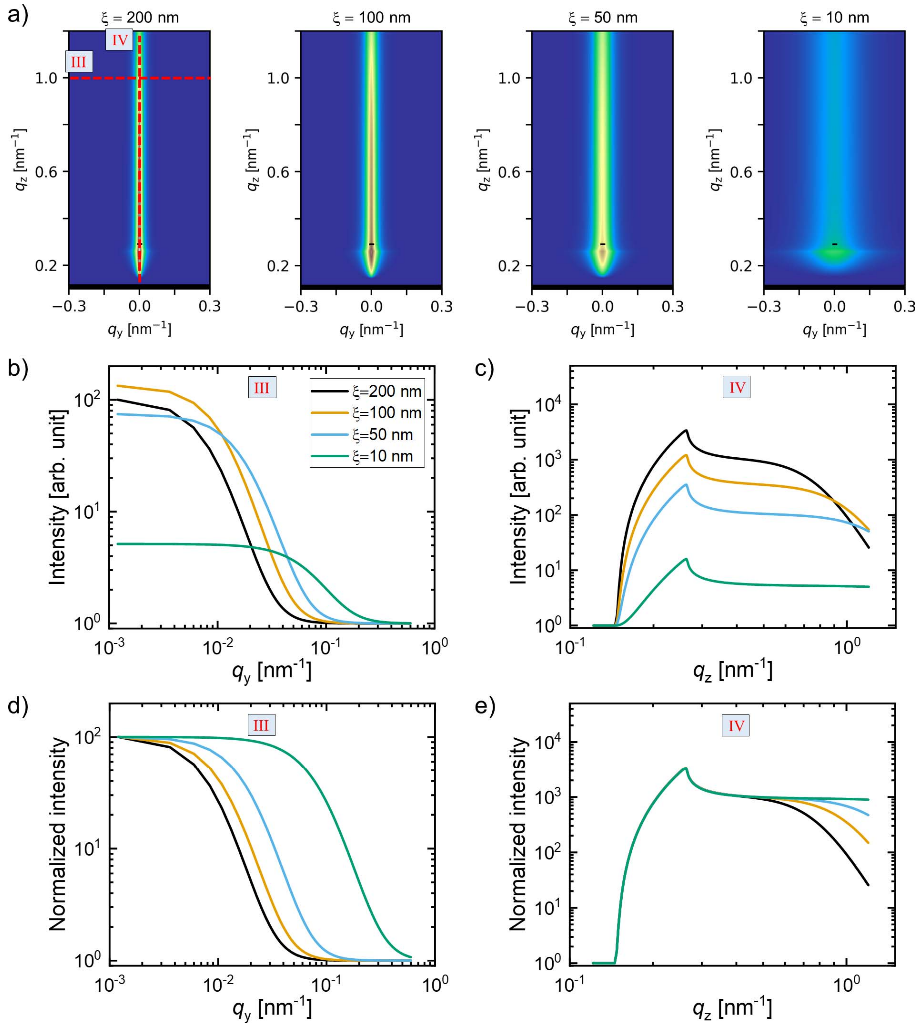

Figure S5 (a) Simulated 2D GISAXS patterns of bare substrates having lateral correlation lengths ξ of 200 nm, 100 nm, 50 nm and 10 nm (left to right). All other parameters in the simulations are the same as in Table S1, i.e., σ_{rms} = 0.1 nm and H = 1.0. (b, c) Linecuts III (b) and IV (c) of the patterns in (a). (d, e) Same linecuts as in (b) and (c), but normalized to the highest intensity of the linecuts at ξ = 200 nm. Note that the normalization factors in (d) and (e) are different to ensure maximum overlap of the respective curves.

The effect of H_{sub} on the 2D GISAXS patterns of the bare substrate and the corresponding linecuts III and IV is shown for $\sigma_{rms,sub}$ = 0.1 nm and ξ_{sub} = 200 nm in Figure S6. The value of H_{sub} affects the 2D GISAXS patterns overall only weakly, but it defines the steepness of the intensity decay at large q-values: With decreasing H_{sub}, the decays in the horizontal linecut III (Figure S6b and d) and the vertical linecut IV (Figure S6c and e) become less steep.

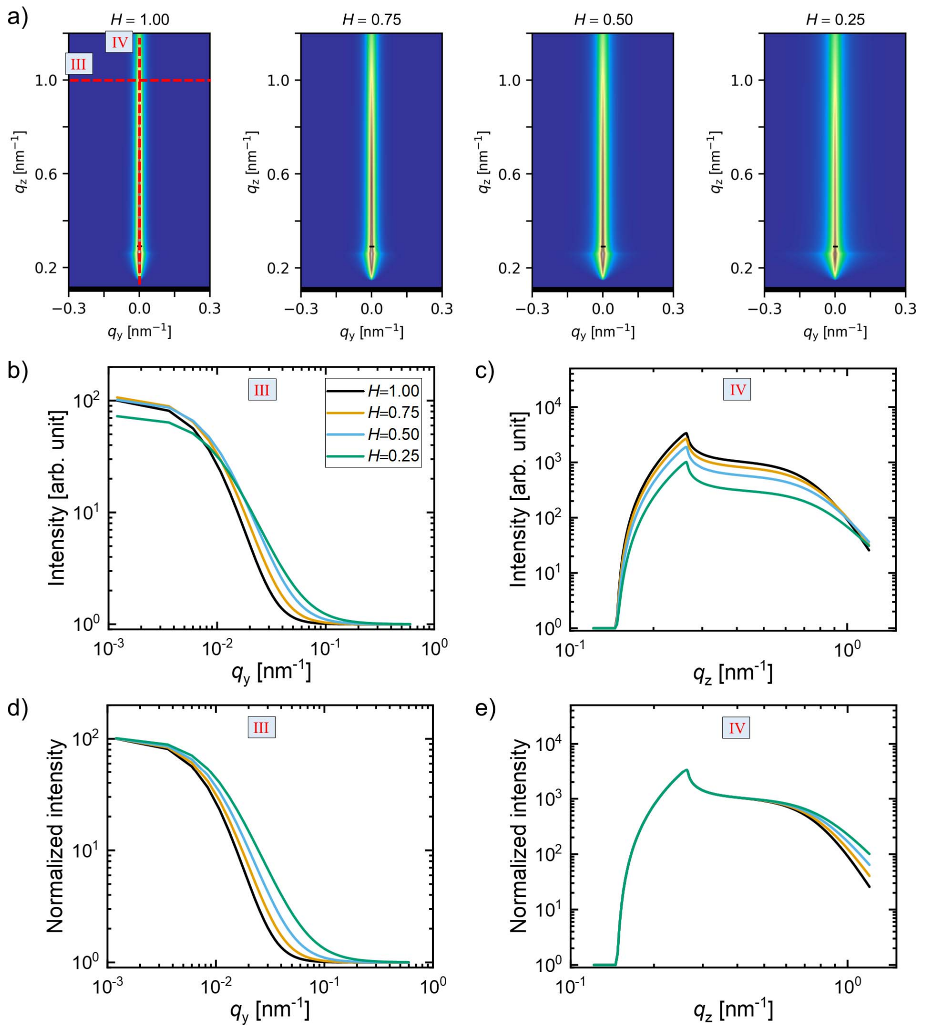

Figure S6 (a) Simulated 2D GISAXS patterns of bare substrates having Hurst parameters, *H*, of 1.0, 0.75, 0.50 and 0.25 (left to right). All other parameters in the simulations are the same as in Table S1, i.e., σ_{rms} = 0.1 nm and ξ = 200 nm. (b, c) Linecuts III (b) and IV (c) of the patterns in (a). (d, e) Same linecuts as in (b) and (c), but normalized to the highest intensity of the cuts at *H* = 1.00. Note that the normalization factors in (d) and (e) are different to ensure maximum overlap of the respective curves.

S4. Additional information for the example

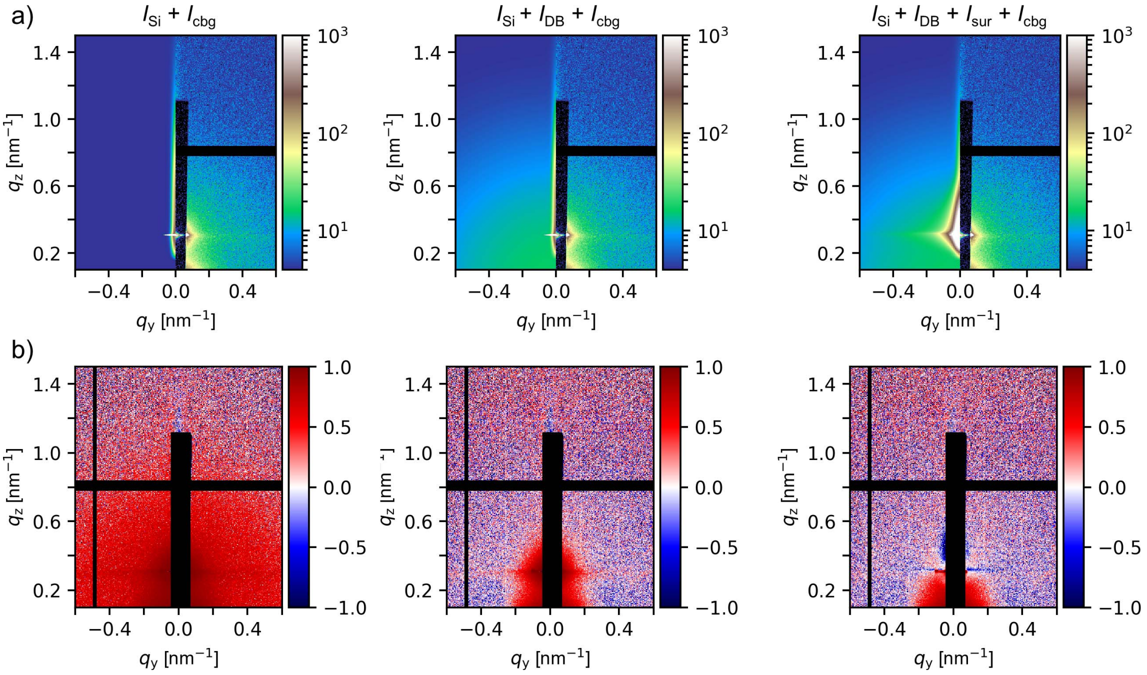

Figure S7 (a) Experimental ($q_y > 0$) and simulated ($q_y < 0$) 2D GISAXS pattern of the bare substrate measurement described in Figure 9. The three cases of a single surface with low surface roughness, the addition of direct beam scattering from a spherical object and the further addition of a surface with high roughness are shown from left to right and are indicated at the top. Different to Figure 9, the detector gap (horizontal black rod) and the beam stop (vertical black rod) are not masked in the experimental pattern. In the simulated pattern, these features are absent, and no mask is applied. This way, the specularly diffuse scattering near $q_y = 0$ is observable in the simulation. (b) Residual plots (see equation 3 in the main text) of experimental and simulated patterns shown directly above. In the residual images, a mask covering the beam stop and detector gaps is applied.

Table S2 Parameters used in the simulation of the bare substrate in Figure 9 and Figure S7.

Instrumental parameters	Wavelength, λ	0.155 nm
	Sample-detector distance, *sdd*	1951 mm
	Detector dimension	981 × 1043 pixels
	Pixel size, p_s	172 µm
	Direct beam position	627.0, 187.6
	Horizontal detector resolution	500 µm
	Vertical detector resolution	20 µm
	Incident angle, α_i	0.22°
Si substrate (I_{Si})	Beam intensity, A_{sample}	8.33×10^{11}
	Dispersion coefficient, δ_{sub}	7.68×10^{-6}
	Absorption coefficient, β_{sub}	177×10^{-9}
	Root-mean-square roughness, $\sigma_{rms,sub}$	0.1 nm
	Lateral correlation length, ξ_{sub}	200 nm
	Hurst parameter, H_{sub}	1.0
Al surface (I_{sur})	Beam intensity, A_{sur}	1.98×10^{11}
	Dispersion coefficient, δ_{sur}	8.58×10^{-6}
	Absorption coefficient, β_{sur}	159×10^{-9}
	Root-mean-square roughness, $\sigma_{rms,sur}$	5.0 nm
	Lateral correlation length, ξ_{sur}	60 nm
	Hurst parameter, H_{sur}	0.5
Direct beam (I_{DB})	Beam intensity, A_{DB}	10^{11}
	Sphere radius, r_{sph}	2.88 nm
Other	Constant background, I_{cbg}	4.0
	Noise scale factor, γ_{noise}	0.0

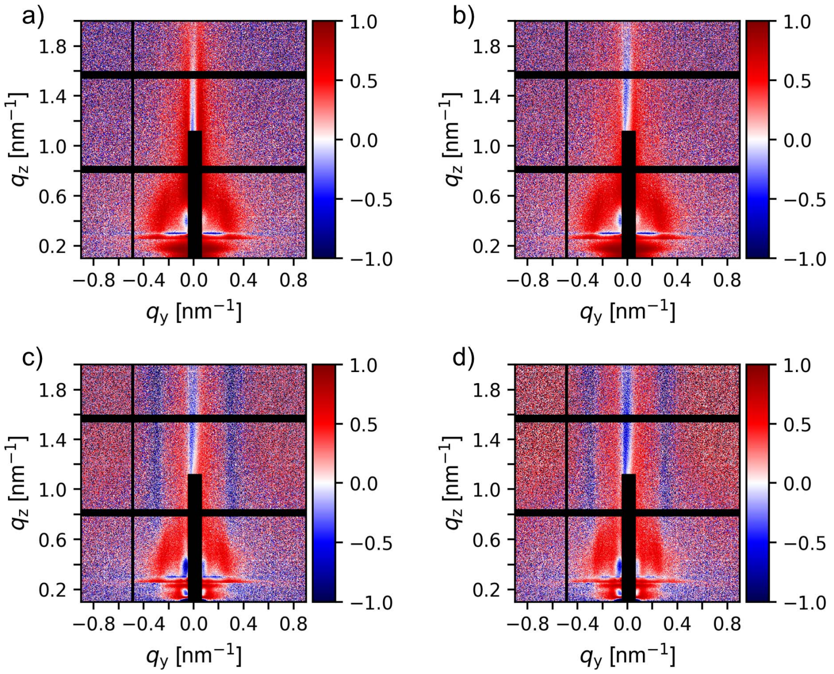

Figure S8 Residual plots (see equation 3 in the main text) of experimental and simulated patterns of the example film at step 1 (a), step 2 (b), step 3 (c) and step 4 (d).

Table S3 Parameters used in the simulation of the example film in Figure 10.

	Instrumental parameters	Wavelength, λ	0.155 nm
		Sample-detector distance, *sdd*	1951 mm
		Detector dimension	981 × 1043 pixels
		Pixel size, p_s	172 µm
		Direct beam position	627.0, 187.6
		Horizontal detector resolution	500 µm
		Vertical detector resolution	20 µm
		Incident angle, α_i	0.195°
		Beam intensity, A_{sample}	6.1×10^{12}
Step 1	Si substrate	Dispersion coefficient, δ_{sub}	7.68×10^{-6}
		Absorption coefficient, β_{sub}	177×10^{-9}
		Root-mean-square roughness, $\sigma_{rms,sub}$	0.1 nm
		Lateral correlation length, ξ_{sub}	200 nm
		Hurst parameter, H_{sub}	1.0
	Al surface (I_{sur})	Beam intensity, A_{sur}	8.56×10^{11}
		Dispersion coefficient, δ_{sur}	8.58×10^{-6}
		Absorption coefficient, β_{sur}	159×10^{-9}
		Root-mean-square roughness, $\sigma_{rms,sur}$	5.0 nm
		Lateral correlation length, ξ_{sur}	60 nm
		Hurst parameter, H_{sur}	0.5
	Direct beam (I_{DB})	Beam intensity, A_{DB}	6×10^{10}
		Sphere radius, r_{sph}	2.88 nm
	Other	Constant background, I_{cbg}	2.0
		Noise scale factor, γ_{noise}	0.0
Step 2	Film	Dispersion coefficient, δ_{film}	4.4×10^{-6}
		Absorption coefficient, β_{film}	8.3×10^{-9}
		Root-mean-square roughness, $\sigma_{rms,film}$	0.6 nm
		Lateral correlation length, ξ_{film}	100 nm
		Hurst parameter, H_{film}	0.5
		Film thickness, *t*	257 nm
		Vertical cross-correlation, $\xi_\perp$	0 nm (uncorrelated)
Step 3	Cylindrical protrusions	Scale factor, I_{pro}	8×10^{-3}
		Radius, r_{pro}	6 nm
		Height, h_{pro}	1 nm
		Average spacing, d_{pro} assuming a radial paracrystal	20 nm
		Width of Gaussian distribution of the paracrystal, ω_{pro}	5 nm

	Cylindrical aggregates	Scale factor, I_{agg}	3×10^{-5}
		Radius, r_{agg}	30 nm
		Width of Gaussian distribution of the radius, $\sigma_{r,agg}$	6 nm
		Height, h_{agg}	30 nm
		Width of Gaussian distribution of the height, $\sigma_{h,agg}$	6 nm
Step 4	Cylindrical inner structure	Scale factor, I_{cyl}	5.54×10^{-4}
		Radius, r_{cyl}	5.93 nm
		Width of Gaussian distribution of the radius, $\sigma_{r,cyl}$	1.19 nm
		Height, h_{cyl}	10 nm
		Average spacing, d_{cyl} assuming a radial paracrystal	21.5 nm
		Width of Gaussian distribution of the paracrystal, ω_{cyl}	3.8 nm
